# Benchmarking physics-inspired machine learning models for transition metal complexes with diverse charge and spin states

**DOI:** 10.1039/d5dd00571j

**Published:** 2026-04-28

**Authors:** Yuri Cho, Ksenia R. Briling, Yannick Calvino Alonso, Ruben Laplaza, Clemence Corminboeuf

**Affiliations:** a Laboratory for Computational Molecular Design, Institute of Chemical Sciences and Engineering, École Polytechnique Fédérale de Lausanne (EPFL) Lausanne Switzerland clemence.corminboeuf@epfl.ch; b National Centre for Computational Design and Discovery of Novel Materials (MARVEL), École Polytechnique Fédérale de Lausanne (EPFL) Lausanne Switzerland; c National Centre for Competence in Research–Catalysis (NCCR–Catalysis), École Polytechnique Fédérale de Lausanne (EPFL) Lausanne Switzerland

## Abstract

Physics-inspired machine learning (ML) models can be categorized into two classes: those relying solely on three-dimensional structure and those incorporating electronic information. In this work, we benchmark both classes for predicting quantum-chemical properties of transition metal complexes with diverse charge and spin states, using three complementary datasets. The evaluated methods include molecular representations (SLATM, FCHL, SOAP, and SPA^H^M family) combined with kernel ridge regression, as well as geometric deep learning models (MACE and 3DMol). We examine how the inclusion of electronic information affects predictive accuracy across datasets and target properties. Models that incorporate electronic information consistently outperform purely structure-based models for properties whose distributions are strongly governed by electronic characters, such as spin-splitting energies and frontier orbital energies. In contrast, structure-only models perform well for predicting the HOMO–LUMO gap and dipole moment magnitude, whose distributions are relatively insensitive to electronic characteristics. Geometric deep learning models with charge and spin embeddings (MACE-QS and 3DMol-QS) show the highest overall accuracy, with 3DMol offering the best computational efficiency among the tested models. These results clarify when geometric information is sufficient and when incorporating electronic information becomes essential, providing practical guidance for selecting effective physics-based ML models for transition metal complexes.

## Abstract 2

We benchmark two classes of physics-inspired machine learning models for predicting quantum-chemical properties of transition metal complexes using three complementary datasets.

## Graphical abstract

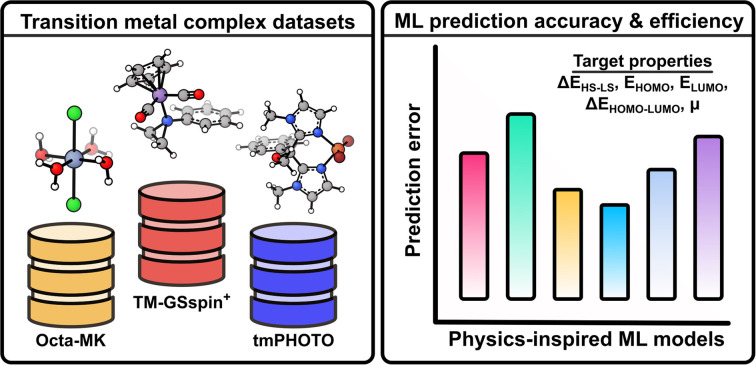

## 1 Introduction

Machine learning (ML) models grounded in physical principles^1^ have emerged as powerful and widely adopted methods for predicting molecular properties in chemistry and materials science.^2–21^ Although their physical origins and architectures vary, these models share the same fundamental inputs: sets of nuclear charges {*Z*_*I*_} and Cartesian coordinates {**R**_*I*_}. This is analogous to the molecular Hamiltonian in quantum mechanics, which determines a molecule's energy and other properties based on the atomic types and positions in three-dimensional space (assuming a neutral singlet ground state). However, beyond structural information, electronic features arising from total charge and spin are also essential for accurate prediction, especially in datasets containing molecules with diverse charge and spin states. Depending on whether and how electronic information is incorporated, existing models can be broadly categorized into two groups: (1) those that rely solely on three-dimensional structures and (2) those that incorporate both structural and electronic information.

The first group includes molecular representations in which structural information is transformed into fixed-size vectors by reflecting known physical laws. Examples include Coulomb matrix,^2,3^ Bag of Bonds,^4^ the Spectrum of London and Axilrod–Teller–Muto potential (SLATM),^5^ Faber–Christensen–Huang–Lilienfeld (FCHL18,19),^6,7^ Smooth Overlap of Atomic Positions (SOAP),^8,9^ Many-Body Tensor Representation (MBTR),^10^ and convolutional Many–Body Distribution Functionals (cMBDF).^11,12^ Typically used with kernel methods such as kernel ridge regression (KRR) or Gaussian process regression, these methods have been successfully applied to the prediction of thermodynamical and quantum properties, including atomization energies, enthalpies of formation, heat capacities, and single-point energies.^2–12^ In parallel, geometric deep learning models learn symmetry-aware representations directly from geometric data and have been applied to the prediction of energies, forces, and other properties.^13–19,21^ Representative architectures include SchNet,^14^ PhysNet,^15^ GemNet,^16^ SpookyNet,^17^ EGNN,^18^ MACE,^22,23^ NequIP,^19^ Equiformer,^21,24^ eSEN,^25^ and UMA.^26^ Although most models assume neutral singlet systems, some, such as SpookyNet, MACE, and UMA, allow explicit charge and spin inputs. In addition to molecular property prediction, such models have also been extended to reaction property prediction. A recent example from some of us is 3DReact,^27^ which predicts reaction activation energies based on three-dimensional structures of reactants and product, along with atom-mapping information.

The second category of models incorporates both structural and electronic information, obtained from quantum-mechanical operators and calculations. Representations include the Spectrum of Approximated Hamiltonian Matrices (SPA^H^M),^28,29^ introduced by some of us, the localized orbital-based FJK representation,^30^ the Matrix of Orthogonalized Atomic Orbital Coefficients (MAOC),^31^ and the Molecular Orbital Decomposition and Aggregation (MODA).^32^ Deep learning models follow a similar strategy by embedding electronic information into neural network architectures. MOB-ML^33^ integrates molecular orbital features derived from Hartree–Fock, while ML-EHM^34^ is based on the extended Hückel method. The OrbNet family^35^ advances this paradigm by featurizing symmetry-adapted atomic orbitals and training graph neural networks (GNNs): OrbNet-Equi introduces equivariance,^36^ OrbNet-Spin adds spin-polarized features,^37^ and OrbitAll^38^ utilizes spin-polarized orbital features combined with SE(3)-equivariant GNNs. Natural quantum graphs (NatQGs)^39^ also integrate geometric and electronic features derived from natural bond orbital analyses and train GNNs to predict quantum properties.

A major advantage of representations and models that encode electronic information is their ability to distinguish charge and spin states, which purely structure-based methods cannot achieve when vertical geometries are used. Consequently, they often outperform structure-only models in predicting quantum-chemical properties across datasets with diverse charge and spin multiplicities. For example, eigenvalue-based SPA^H^M^28^ effectively differentiates spin and charge states in datasets such as QM7 ^2,40^ augmented with vertical radical cations and spin- and charge-diverse L11,^41^ and its local successors achieve strong transferability to photoactive systems.^29,42^ Similarly, MAOC^31^ and MODA^32^ predict properties of organic radicals, including single-point energies, frontier orbital energy levels, or magnetic couplings, while the FJK representation^30^ has been validated on larger datasets such as QM9 ^43^ and LIBE,^44^ and in Δ-ML frameworks.^45^ Among deep learning models, OrbNet-Equi^36^ outperforms structure-only representations on neutral closed-shell systems in QM9, while the latest OrbitAll^38^ achieves superior accuracy and transferability across charged and open-shell species in the QM9star dataset.^46^

However, comparative assessments of physics-inspired ML models have so far focused mainly on organic molecules,^28–32,36,38^ leaving their robustness for larger and more complex systems, particularly transition metal (TM) complexes, underexplored. Existing ML studies on TM complexes have relied mainly on graph-based descriptors. For instance, Kulik and collaborators introduced revised autocorrelations (RACs),^47^ heuristic descriptors encoding features such as nuclear charge, electronegativity, and topology. Combined with neural networks, kernel methods, or GNNs, RACs have been used to predict properties of octahedral complexes, including ground-state spin, spin-splitting energies, frontier orbital energies, redox potentials, and multireference character.^47–55^ More recently, many-body expansion representations were proposed to capture metal-centered interactions at higher orders.^56^ NatQGs, developed by Kneiding *et al.*,^39^ integrate geometric and electronic information derived from natural bond orbital analysis and have been benchmarked on tmQM.^57^ Additional benchmarks with different GNNs were also performed on tmQM and its recomputed variant, tmQM_ωB97MV.^58^ Despite these advances, physics-based representations and models have not yet been systematically assessed for TM complexes, which feature broad variations in charge, spin, and coordination environment. A rigorous evaluation is therefore needed to determine how the inclusion of electronic information improves predictive accuracy relative to models that rely solely on structures.

In this work, we systematically evaluate the performance of a selection of physics-inspired ML models in predicting the molecular properties of TM complexes. Our evaluation spans three benchmark datasets of varying sizes and diversity, encompassing differences in metal identity, total charge, and spin distributions. Target properties include spin-splitting energy, highest occupied molecular orbital (HOMO) energy, lowest unoccupied molecular orbital (LUMO) energy, HOMO–LUMO gap, and dipole moment. Both purely structure-based and quantum-informed representations are evaluated using KRR, revealing how electronic information influences the predictive accuracy for across different datasets and target properties. This study also introduces and assesses 3DMol, a molecular variant of our 3DReact geometric deep learning model^27^ originally developed for reaction properties. The performance of 3DMol on molecular properties is compared to the MACE.^22,23^ We further explore potential improvements of deep learning models by evaluating their variants that incorporate charge and spin embeddings. Overall, this study identifies the most effective physics-based ML approaches for TM complexes and clarifies how dataset characteristics and property types guide the choice between structure-only and electronically informed models.

## 2 Datasets

To benchmark model performance in predicting the properties of TM complexes, we use three datasets: TM-GSspin^+^,^59^ tmPHOTO,^57,60^ and Octa-MK (the name we use within this work to refer to the dataset from Meyer *et al.*^56^).

Although all three comprise mononuclear TM complexes with DFT-computed properties, they differ in their curation, dataset size, distributions of metal centers and molecular charges, geometry-optimization methods, and spin-state treatment. An overview of these differences is summarized in Table 1. Since our goal is to evaluate physics-inspired ML models on the property definitions native to each benchmark set rather than enforce methodological uniformity, we therefore train and assess models independently on each dataset as originally curated. This design choice also reflects realistic, application-specific workflows in which data are produced using different in-house pipelines.

**Table 1:** Overview of the benchmark datasets. Δ*E*_HS–LS_ denotes the spin-splitting energy, defined as the energy difference between the high-spin (HS) and low-spin (LS) states. CN refers to the coordination number, and gap indicates the HOMO–LUMO gap. Details of the DFT functionals and basis sets appear in each dataset subsection. Metal oxidation states are not specified for tmPHOTO

Dataset	Octa-MK	TM-GSspin^+^	tmPHOTO
# Complexes	1806	2260	4268
# Unique elements	13	18	25
Metals (oxidation states)	Cr, Mn, Fe, Co (II, III)	Cr, Fe, Ni (0, II, III); Mn, Co (I, II, III)	3d: Fe, Ni, Cu, Zn; 4d: Ru, Pd, Ag, Cd; 5d: Re, Ir, Pt, Au, Hg
Coordination geometry	Octahedral only	Geometries with CN 2–8 or haptic ligands	
Data curation	Bottom-up	Top-down (extracted from molecular crystals)	
Molecular charge	−2 to +3	−5 to +4	−1 to +1
Spin state for computations	LS and HS	Ground-state spin	Singlet
Geometry optimization	DFT at LS and HS	DFT at LS, hydrogens only	GFN2-xTB at singlet
DFT-computed properties	Adiabatic Δ*E*_HS–LS_, HOMO, LUMO, gap	Vertical Δ*E*_HS–LS_, HOMO, LUMO, gap, dipole moment	HOMO, LUMO, gap, dipole moment

**Fig. 1:**
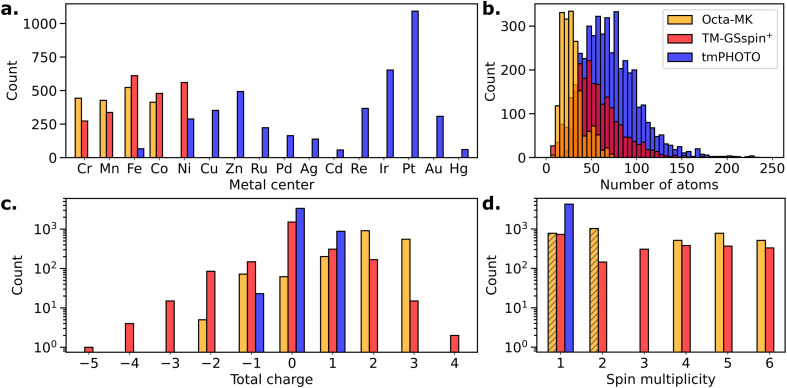
Distribution of key characteristics across the benchmarking datasets: Octa-MK (orange), TM-GSspin^+^ (red), and tmPHOTO (blue). (a) Metal centers, (b) number of atoms, (c) total molecular charges, (d) spin multiplicities used in the computations. For Octa-MK, low-spin (hatched; singlets or doublets) and high-spin (unhatched; quartets, quintets, or sextets) computations were performed for each complex.

Fig. 1 illustrates the distribution of key characteristics, including the identity and frequency of metal centers, the number of atoms, and the molecular charges and spin states used in the computations. These datasets are selected to complement one another and address their respective limitations. TM-GSspin^+^ and Octa-MK cover 3d TMs with identified or assigned oxidation states, whereas tmPHOTO includes 3d, 4d, and 5d metals but lacks oxidation state information. They also exhibit different distributions in total charge and molecular size. In terms of charge diversity, TM-GSspin^+^ spans the widest range of total molecular charges, followed by Octa-MK, while tmPHOTO shows the narrowest distribution, being dominated by neutral or ±1 charged complexes. Conversely, when considering molecular size, tmPHOTO encompasses the broadest range of complex sizes, including the largest systems, whereas Octa-MK primarily consists of smaller octahedral species, with TM-GSspin^+^ occupying an intermediate range.

In terms of spin states, 3d TM complexes with d-electron configurations ranging from d^4^ to d^8^ can adopt different spin states depending on the nature of the metal center and its coordination environment. Reflecting this, Octa-MK provides both low-spin and high-spin optimized geometries, making it suitable for benchmarking spin-state dependencies in geometries. Meanwhile, TM-GSspin^+^ offers the advantage of providing properties computed at the ground-state spin, with the ground state determined using DFT. In contrast, the computed properties in tmPHOTO are restricted to singlet states, which can be limiting, especially for 3d metals that often possess higher ground-state spins.

In terms of chemical diversity, Octa-MK covers a relatively narrow chemical space, focusing only on octahedral complexes with a smaller variety of metals and ligand sizes compared to the other two datasets. Conversely, TM-GSspin^+^ and tmPHOTO encompass a much broader range of coordination geometries, ligand types, and metal centers, as their structures are extracted from crystallographic data covering diverse structural motifs and coordination environments. Among them, tmPHOTO is the largest dataset evaluated in this work, including the widest range of metals and unique elements.

### 2.1 TM-GSspin^+^

TM-GSspin^+^, curated in this work, is an extended version of TM-GSspin,^59^ which was originally constructed to train a ground-state spin prediction model for 3d TM complexes. It expands the original collection of 2063 Cr, Mn, Fe, Co, and Ni complexes with diverse coordination geometries by adding 280 additional complexes featuring haptic ligands, which were available in the SI of the previous work.^59^ Out of 2343 complexes, 71 complexes were removed due to the presence of rarely occurring elements appearing in fewer than 1% of the original dataset (Table S1 for details). Additionally, 6 complexes were excluded due to discrepancies between the chemical formula in the crystallographic information file and the actual crystal structure, specifically missing hydrogen atoms.

Initial structures of the complexes were extracted from molecular crystals reported in the Cambridge Structural Database (CSD)^61^ using 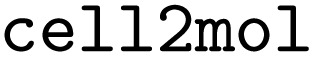 version 1.1.0.^62^ The structures were then refined by optimizing the positions of the hydrogen atoms, while heavy atom coordinates were constrained to their experimental crystal structures. This geometry optimization was performed at the B3LYP*-D3(BJ)^63,64^/def2-SVP^65^ level in the lowest spin state (singlet for even-electron systems and doublet for odd-electron systems). Subsequently, single point computations were carried out at the B3LYP*-D3(BJ)/def2-TZVP level for all accessible spin states, and the spin multiplicity with the lowest energy was assigned as the ground-state spin. Vertical spin-splitting energies were determined as the energy difference between the high-spin (HS) and low-spin (LS) states, computed at the same geometry, where no additional geometry optimization was performed for each spin state. All computations using B3LYP*^66,67^ were performed using Gaussian09 (revision D.01)^68^ and their reliability—in terms of chemical accuracy for DFT spin-splitting energetics and spin contamination in TM complexes—was validated in our previous work.^59^

Following this, single point energy computations at the ground state spin were performed at the TPSSh^69,70^-D3(BJ)/def2-TZVP level using ORCA version 5.0.3 ^71^ to obtain additional properties, such as HOMO, LUMO, HOMO–LUMO gap, and dipole moment. Additionally, 6 complexes were excluded due to spin contamination in the TPSSh computations, where the 〈*Ŝ*^2^〉 value deviated from the exact value of *S*(*S* + 1) by more than 0.1 for doublets and more than 0.2 for higher spin states.^72,73^

The final TM-GSspin^+^ dataset used in this work comprises 2260 complexes, with properties computed at their ground-state spin and vertical spin-splitting energies for each complex.

### 2.2 tmPHOTO

tmPHOTO, curated by Kevlishvili *et al.*,^60^ is a subset of tmQM reported by Balcells *et al.*^57^ tmQM comprises 86 665 mononuclear TM complexes extracted from CSD, featuring 30 TMs (spanning the 3d, 4d, and 5d series from groups 3 to 12) with total molecular charges ranging from −1 to +1. The geometries of tmQM complexes were optimized at the GFN2-xTB^74^ level, and single point computations were performed at the TPSSh-D3(BJ)/def2-SVP level at the singlet state to provide HOMO, LUMO, HOMO–LUMO gap, dipole moment, and other properties.

tmPHOTO was constructed using natural language processing to link tmQM complexes to application based on information extracted from manuscript titles and abstracts.^60^ In their work, tmPHOTO focuses on TM complexes relevant to photophysical applications and was further expanded *via* structural mapping,^60^ ultimately growing to 4599 complexes. However, when considering only entries with unique CSD ref codes from the original tmQM, we identify 4500 complexes. To ensure consistency with the procedure used in TM-GSspin^+^, we excluded 232 complexes containing rarely occurring elements—defined as those comprising fewer than 1% of the original dataset size (Table S1). After filtering, the final tmPHOTO used in this work consists of 4268 complexes.

### 2.3 Octa-MK

Octa-MK, curated by Meyer *et al.*,^56^ is assembled from six previous studies,^47–49,51,53,54^ focusing on octahedral TM complexes of four 3d metals (Cr, Mn, Fe, Co) with oxidation states II and III. Initial geometries were generated using 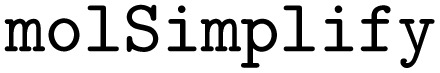,^75,76^ which employs 
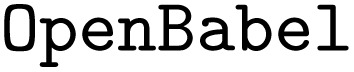
^77^ as a backend for ligand structure generation, by combining metal centers with a predefined ligand list. Meyer *et al.*^56^ curated complexes with DFT-optimized geometries in both LS and HS states, along with their corresponding computed properties. All DFT calculations used the B3LYP^78–80^ functional with the LACVP* basis set (LANL2DZ effective core potential^81^ for iodine and TMs, and 6-31G*^82^ for all other atoms). After excluding complexes with positive HOMO energies, significant spin contamination, or large deviations from expected octahedral geometry, the final dataset contains 1806 LS/HS pairs spanning 107 unique ligands (72 monodentate, 34 bidentate, and 1 tetradentate).

## 3 Machine learning models

### 3.1 Molecular representations

We examine a set of physics-based molecular representations, divided into two categories: (a) SLATM,^5^ FCHL,^6,7^ and SOAP,^8^ which rely solely on three-dimensional structures; and eigenvalue SPA^H^M^28^ and its extension SPA^H^M(a,b),^29^ which are quantum-informed representations that inherently encode spin and charge *via* quantum-mechanical operators. SLATM, FCHL, and SOAP are included as well-established and widely used representations that have demonstrated strong performance across diverse datasets. The SPA^H^M family is included for its superior ability to capture spin, charge and electronic-state differences particularly in charged systems, outperforming purely structure-based representations.

(a) SLATM^5^ is built by concatenating one-, two- and three-body potentials separated into element-specific bags defined by the nuclear charges of the participating atoms. FCHL^6,7^ encodes atomic environments through a two-body term that captures radial distributions and a three-body term that encodes mean distances and angles, both parameterized by the element types of neighboring atoms. In this work, we employ the FCHL19,^7^ the latest version known for its compact representation. SOAP^8^ represents local atomic environments through a local expansion of Gaussian-smeared atomic densities onto orthonormal functions derived from spherical harmonics and radial basis functions.

Eigenvalue SPA^H^M (ε-SPA^H^M)^28^ is a global representation built from occupied-orbital eigenvalues of a light-weight one-electron Hamiltonian,^83^ typically used as initial guess for self-consistent field quantum-chemical computations. SPA^H^M(a)^29^ and SPA^H^M(b)^29^ are local and transferable extension of ε-SPA^H^M, utilizing one-electron density matrices on the same initial-guess to generate fingerprints based on atomic and bond density contributions, respectively.

To ensure consistent comparison across methods, we focus on fixed-size representations compatible with kernel-based methods, specifically KRR due to its efficiency and robustness in modeling nonlinear relationships between representations and target properties. We also examine the purely structure-based cMBDF^11,12^ and two other quantum-informed representations, MODA^32^ and PC3-MAOC.^31^ Their results are summarized in Table S2, as they show lower performance within their respective categories.

### 3.2 Geometric deep learning models

#### 3.2.1 MACE

MACE^22,23^ is a state-of-the-art machine learning force field architecture, which predicts the potential energies and forces in molecules and materials. It is based on equivariant message passing neural networks and introduces a hierarchical message construction scheme grounded in body-order expansion. MACE parametrizes the mapping from atomic positions and chemical elements to the total potential energy by decomposing it into atomic (site) energy contributions, each determined by symmetric, many-body features expressed in a spherical harmonics basis. MACE was selected in this study due to its excellent performance across a wide range of benchmark datasets, from small organic molecules to liquids and solids.^84^

In this work, we train the models from scratch while adopting the hyperparameters of the MACE model with message equivariance order 2 (*L*_max_ = 2) as employed by Kovács *et al.*^85^ for predicting the properties in the QM9 dataset.^43^ QM9 comprises organic molecules with up to nine heavy atoms, provided at equilibrium geometries with zero atomic forces. Because the target properties considered here are intensive energy quantities, the loss function is modified to exclude force terms, and the standard sum-pooling readout, appropriate for extensive quantities such as total potential energy, is replaced by mean pooling to correctly handle intensive targets.

In addition to the equivariant MACE (*L*_max_ = 2), we also evaluate an invariant model (*L*_max_ = 0). The performance of both models in predicting intensive energy targets is summarized in Table S3 in the SI. Since the two models achieve comparable accuracies, we adopt the invariant model for energy prediction due to its lower computational cost.

For dipole moment predictions, we employ the AtomicDipolesMACE architecture with equivariant messages *L*_max_ = 2, which is originally designed to predict dipole moment vectors. Because our target property is the magnitude of the dipole moment, we modify the AtomicDipolesMACE model to compute the magnitude from the predicted vectors and adjust the corresponding loss function accordingly.

#### 3.2.2 3DMol

3DMol (adapted from our 3DReact,^27^ model that uses learned representations of reactants and products to predict reaction properties) is an equivariant message passing neural network based on the tensor field network architecture,^86–88^ designed for single-molecule input.

A molecule is represented as a distance-based graph with hydrogen atoms excluded. Four of the initial atomic features are derived from the molecular structure: effective atom surface and volume, computed with 
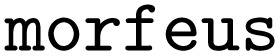
,^89^ the occupied volume, and the number of directly-bonded neighbors. Additionally, the tabulated number of valence electrons and Pauling electronegativity of the element are used as other two initial node features.

The initial features are passed through embeddings and then updated by equivariant convolutional layers defined by spherical harmonics used to construct the filters.^87^ In this work, we use only scalar harmonics (equivalent to *L*_max_ = 0 for MACE) and thus an invariant architecture, since previous works show that enabling *L*_max_ > 0 does not necessary improve prediction of scalar properties.^27^ These convolutional layers output features for each atom in molecule, which are used for property prediction (see Section 3.3).

#### 3.2.3 3DMol-QS and MACE-QS

As 3DMol and MACE rely solely on three-dimensional structures, we also test the variants that incorporate charge and spin embeddings, referred to as 3DMol-QS and MACE-QS, respectively. In these charge and spin embedded versions, the charge and spin values are provided as global scalar inputs, embedded into the model's latent feature space, and subsequently added to the initial node features of each atom.

This approach differs from that of the quantum-informed models (discussed in the previous subsection), which encode electronic information arising from charge and spin implicitly through quantum-mechanical operators (*e.g.*, the guess Hamiltonian). Because the AtomicDipolesMACE architecture does not support charge or spin embeddings, no MACE-QS variant is available for dipole moment prediction. The implementation of charge and spin embeddings follows the approach used in the MACE-OMol-0 foundation model, which was trained on the OMol25 dataset.^90^

### 3.3 Global and local variants

Molecular representations encode either the entire molecule or individual atoms. This work evaluates both variants when available. The local variant refers specifically to the atomic representation of the TM center, as each complex contains a single metal atom, while the global representation is obtained by summing all atomic vector within the molecule. Only the global variant is available for ε-SPA^H^M.

For 3DMol, the global variant sums atomic features into a single representation vector, while the local variant predicts properties using only the metal atom features. For MACE and MACE-QS, only the global variant is implemented in this work.

### 3.4 Performance evaluation and hyperparameters

We evaluate model performance using 10-fold cross-validation (CV), reporting the average MAE over the ten test sets. To ensure consistency, all models and target properties within each dataset use identical training and test splits. For the deep learning models, each training set is further divided into training and validation (8 : 1), resulting in 80/10/10 splits.

For Octa-MK, additional considerations are required. The original study by Meyer *et al.*^56^ employed a 80/20 train/test split while ensuring coverage of unseen ligand variations. In this work, we use the full dataset of 1806 complexes and perform 10-fold CV. Because each complex provides both LS and HS geometries with the corresponding frontier orbital energies, the dataset contains 3612 geometries and 3612 reference labels per property type. When predicting frontier orbital energies, the LS and HS geometries of the same complex are placed in the same test fold to avoid information leakage, while still randomly splitting the complexes themselves.

KRR hyperparameters are selected through 5-fold CV on each training set, with the parameters defined as 
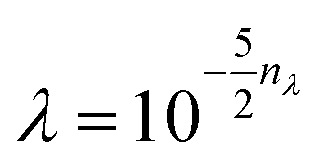
, *σ* = 10^*n*_*σ*_/2^, 
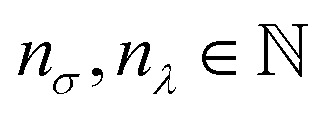
. The regularization parameter *λ* is optimized over a fixed grid 0 ≤ *n*_*λ*_ ≤ 4, while the kernel width *σ* is optimized on an adaptive grid starting from 0 ≤ *n*_*σ*_ ≤ 12.

For the molecular representations, we use the default parameters as defined in the GitHub repositories released by the original developers, except for SOAP. SLATM and FCHL are generated using 
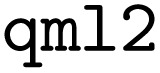
,^91^ and SPA^H^M family is generated using 
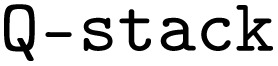
.^92^ SOAP is generated using 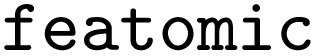,^93^ adopting the key parameters values reported in Lopanitsyna *et al.*,^94^ which used SOAP features to build a potential capable of describing 25 TMs. Parameter details are given in Tables S4–S7 in the SI.

The 3DMol is trained using the Adam optimizer,^95^ reducing the learning rate by 40% after 60 epochs without validation improvement. Training proceeds for up to 512 epochs with early stopping after 150 stagnant epochs. The model achieving the lowest validation MAE is used for testing. All 3DMol computations employ the invariant architecture and exclude hydrogen atoms from the graphs. The hyperparameters are optimized for each dataset, property, and local or global variant using Bayesian search as implemented in Weights & Biases,^96^ with the search space provided in Table S8. For TM-GSspin^+^ and tmPHOTO, the first split from the 10-fold CV is used for hyperparameter optimization. For Octa-MK, the optimal hyperparameters are obtained using the 80/20 train/test split of the original study,^56^ further divided into a 60/20/20 training/validation/test split. The parameters giving the lowest validation MAE after 128 epochs are selected for each dataset (Tables S9–S11). The unprocessed 3DMol results are available at https://wandb.ai/equireact/3dmol-TMC-benchmark.

The MACE models for energy prediction use the same hyperparameters as those reported by Kovács *et al.*,^85^ employing 256 uncoupled channels. The equivariant variant sets the message equivariance order to *L*_max_ = 2, whereas the invariant variant uses *L*_max_ = 0. Training proceeds for up to 650 epochs with a batch size of 2. The initial learning rate is 10^−3^, and a scheduler reduces the learning rate when the validation loss does not improve for five consecutive epochs. Early stopping is triggered after fifteen stagnant epochs. Stochastic weight averaging (SWA) is enabled from epoch 450, and an exponential moving average (EMA) of the weights with decay 0.999 is maintained throughout training to improve stability and generalization. For dipole moment prediction, we employ the same hyperparameters but replace the model with AtomicDipolesMACE using equivariant messages (*L*_max_ = 2), and SWA is not applied. Hyperparameter used for all MACE models are listed in Table S12.

## 4 Results and discussion

The target properties evaluated across the three datasets include spin-splitting energies, frontier orbital energies, their gap, and dipole moment magnitudes, all of which are central to understanding reactivity, stability, magnetism, and spectroscopic behavior. For each property, we first examine its distribution to clarify how electronic information shapes the overall spread of values and influences model performance. We further assess predictive accuracy for the HOMO and HOMO–LUMO gaps within subsets grouped by total molecular charges. Finally, we compare the computational efficiency of the models.

### 4.1 Spin-splitting energy

We use TM-GSspin^+^ and Octa-MK to assess model performance in predicting spin-splitting energies, defined as the energy difference between the HS and LS states. TM-GSspin^+^ provides vertical spin-splitting energies, since LS geometries, optimized only for hydrogen atoms, are employed to compute single point energies for all accessible spin states of a given d-electron configuration (*e.g.*, d^4^ Cr(ii): singlet, triplet, quintet; d^5^ Mn(ii): doublet, quintet, sextet). Octa-MK, by contrast, provides adiabatic spin-splitting energies as it contains independently optimized LS and HS geometries. However, it includes only these two spin states (*e.g.*, d^4^ Cr(ii): singlet, quintet; d^5^ Mn(ii): doublet, sextet) and therefore does not identify ground-state spins for d^4^ to d^6^ complexes.

**Fig. 2:**
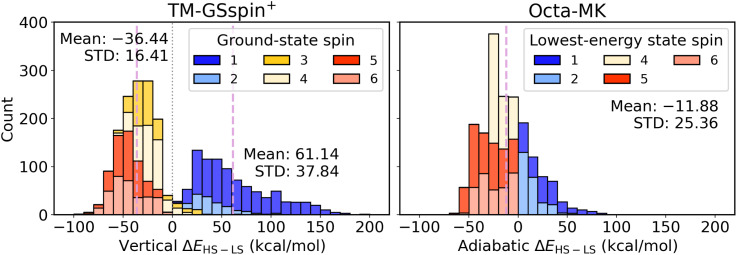
Stacked histograms of spin-splitting energies, Δ*E*_HS–LS_, for the TM-GSspin^+^ (left) and Octa-MK (right) datasets. Colors denote the spin multiplicity of the lowest-energy state among the spin states considered in each dataset. For TM-GSspin^+^, the dashed lines mark the mean Δ*E*_HS–LS_ values for the two subsets with Δ*E*_HS–LS_ < 0 and Δ*E*_HS–LS_ > 0, and the dotted line marks Δ*E*_HS–LS_ = 0; the corresponding mean and standard deviation (STD) are reported in the panel. For Octa-MK, the dashed line marks the mean Δ*E*_HS–LS_ for the full dataset, and the corresponding mean and STD are reported in the inset.

Fig. 2 displays the spin-splitting energy distributions as stacked histograms, colored according to the spin multiplicity of the lowest-energy state considered in each dataset. LS complexes with singlet or doublet ground states generally show positive values, although a small number of TM-GSspin^+^ complexes with triplet or quartet ground states also exhibit positive spin-splitting energies. The two datasets exhibit clearly different distribution profiles. TM-GSspin^+^ is bimodal, with two distinct peaks near −33 and 37 kcal mol^−1^ and an overall range of −91 to 191 kcal mol^−1^. In contrast, Octa-MK shows a unimodal distribution with a peak near −13 kcal mol^−1^ and a narrower range from −61 to 84 kcal mol^−1^.

The broader distribution of spin-splitting energies in TM-GSspin^+^ reflects its greater chemical diversity, which spans a wider range of ligand environments, coordination geometries, inclusion of Ni centers, and a more extensive set of d-electron configurations. Octa-MK, by contrast, contains d^3^ to d^7^ octahedral complexes with smaller ligands, resulting in a narrower property distribution. Differences in dataset curation also contribute to the distinct shapes. In TM-GSspin^+^, complexes with small energy difference between two low-lying spin states were removed in previous work^59^ to ensure reliable ground-state assignments, reducing the population near zero. A few d^4^ to d^6^ complexes with intermediate-spin ground states remain in the range of −5 to 5 kcal mol^−1^. Octa-MK retains more complexes in this region since no such filtering is applied. It also includes spin-crossover candidates generated by a genetic algorithm,^49^ defined by having small absolute spin-splitting energies.

Although the spin-splitting energy is a global property of the complex, it is often strongly influenced by the metal center because changes in spin state frequently involve the metal d-orbitals. However, depending on the degree of metal–ligand covalency and the electronic nature of the ligands, spin transitions can also involve significant ligand contributions or even become predominantly ligand-centered. To assess the character of the spin transition, we analyze the Hirshfeld spin populations in the LS and HS states of TM-GSspin^+^ using both vertical and adiabatic computations (Fig. S1 and S2 in the SI). Vertical denotes hydrogen-only optimization in the LS state (singlet or doublet) at B3LYP*-D3(BJ)/def2-SVP, followed by TPSSh-D3(BJ)/def2-TZVP single-point computations for all accessible spin states. Adiabatic denotes full-geometry optimization at B3LYP*-D3(BJ)/def2-SVP for each spin state, followed by a TPSSh-D3(BJ)/def2-TZVP single-point computation for the corresponding spin state.

Specifically, we compare the LS-to-HS spin transition in terms of the contribution on the metal center, the cumulative contribution from atoms within 4.5 Å of the metal center, and the contribution from all ligand atoms. The 4.5 Å cutoff corresponds to the smallest distance explored among the representations considered. As demonstrated in Fig. S1 and S2, for the majority of complexes, the spin transition remains largely localized at or near the metal center, although we also observe some complexes in which ligand atoms farther than 4.5 Å from the metal center make a non-negligible contribution. We therefore evaluate both local (metal-centered) and global (whole-complex) variants to assess whether the spin transition is described sufficiently by the metal-centered atomic environment alone or whether more distant ligand contributions must also be included. The only exceptions are ε-SPA^H^M, MACE and MACE-QS, for which only the global variant is considered in this work. Additionally, Fig. S3 compares the vertical and adiabatic spin-splitting energies of TM-GSspin^+^ and shows that they are highly correlated.

**Fig. 3:**
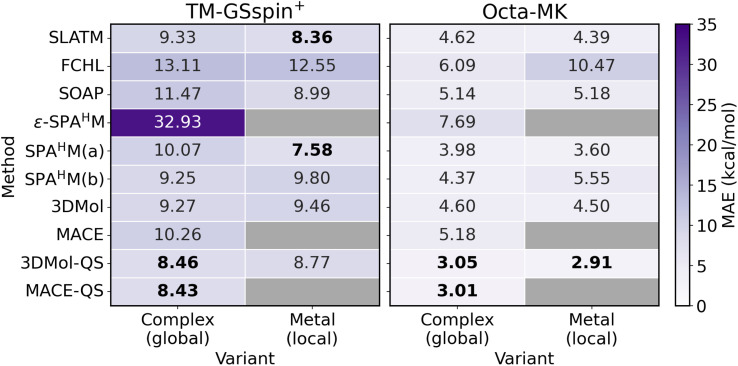
Mean absolute errors (MAEs) of physics-based ML models for predicting spin-splitting energies in TM-GSspin^+^ (left) and Octa-MK (right) datasets using 10-fold cross-validation. For each model, both global (entire complex) and local (metal center) variants are shown where applicable. The best-performing method/variant combinations (considering standard deviation) are highlighted in bold.

Fig. 3 reports the MAEs of the models for predicting spin-splitting energies (Δ*E*_HS–LS_) for TM-GSspin^+^ and Octa-MK, with the corresponding standard deviations provided in Table S13. Since TM-GSspin^+^ exhibits a bimodal distribution, we further assess the performance of the KRR models for each mode separately by performing independent 10-fold CV on the subsets defined by the sign of Δ*E*_HS–LS_, as summarized in Table S14. In addition, Table S15 reports the MAEs from the 80/20 train/test splits of Meyer *et al.*,^56^ together with their results for standard RACs and two- or three-body representations in the original study.

In Fig. 3, among the KRR models based on representations, local SPA^H^M(a) yields the lowest MAEs, with values of 7.58 kcal mol^−1^ for TM-GSspin^+^ and 3.60 kcal mol^−1^ for Octa-MK. Although 3DMol and MACE are not top performers, their charge and spin embedded models achieve the best or comparable accuracy across both datasets. These trends indicate that incorporating electronic information improves the predictive accuracy of spin-splitting energies, whether introduced implicitly through quantum-mechanical operators or explicitly by embedding charge and spin as additional features. A notable exception is ε-SPA^H^M, whose especially poor performance on TM-GSspin^+^ may reflect limitations of the representation itself or dataset-induced bias, as discussed later. However, this behavior is unlikely to stem from a poor initial guess, since all three SPA^H^M variants, ε-SPA^H^M, SPA^H^M(a), and SPA^H^M(b), are derived from the same initial guess based on the DFT-determined ground-state spin configuration, yet produce models with substantially different performance.

When comparing local and global variants on TM-GSspin^+^, their performances are largely similar, with the local variants sometimes showing a small advantage. This suggests that describing the local environment around the metal center is often sufficient for predicting this property and becomes more useful as global variants grow more expensive for larger complexes. In Octa-MK, however, local FCHL performs much worse than its global counterpart, while the other models behave consistently across variants. The poor performance of local FCHL on Octa-MK likely arises from the high symmetry of octahedral metal centers, where metal–ligand distances and angles collapse to a small set of characteristic values. This produces highly simplified radial and angular distributions, preventing the local FCHL19 from distinguishing subtle structural variations and leading to elevated prediction errors.

Because Octa-MK provides both LS and HS optimized geometries, we examine how spin-state dependent structural changes influence the performance of KRR models using these representations (Table S16). With structure-based representations such as SLATM, FCHL, and SOAP, LS optimized geometries consistently yield slightly lower MAEs than HS optimized geometries, regardless of whether the representations are local or global. For the quantum-informed SPA^H^M family, the spin state used to construct the representation must also be specified. When the representation is built using LS states, LS optimized geometries naturally produce lower errors than HS optimized geometries paired with LS states. When each representation instead employs the lowest-energy spin state of the complex, the performance gap between LS and HS optimized geometries becomes smaller, because the representation already encodes the electronically preferred state and is therefore less sensitive to structural differences between the two optimized geometries. Overall, however, the effects of both spin-state-dependent structural changes and the choice of spin state used to construct the quantum-informed representations remain minor for KRR model performance.

Lastly, given the bimodal distribution of spin-splitting energies in TM-GSspin^+^, we further examine the performance of KRR models across subsets defined by the sign of Δ*E*_HS–LS_, corresponding to the two distinct modes. MAEs are computed using two approaches: (i) 10-fold CV on the full dataset, followed by grouping the resulting test set errors by the sign of the Δ*E*_HS–LS_ (Fig. S4 and S5), and (ii) independent 10-fold CV within each subset (Table S14).

For the approach (i), four KRR models are evaluated: two global representations (SLATM and ε-SPA^H^M) and two local representations (aSLATM and SPA^H^M(a)). Fig. S4 reports the MAEs derived from the complexes with Δ*E*_HS–LS_ < 0 and Δ*E*_HS–LS_ > 0, alongside the error rates for predicting the correct HS/LS energetic ordering. Compared to other representations, ε-SPA^H^M produces much larger errors across both Δ*E*_HS–LS_ regimes and yields a significantly higher fraction of predictions with the incorrect sign of Δ*E*_HS–LS_. In addition, Fig. S5 shows parity plots comparing ML-predicted and DFT reference spin-splitting energies for TM-GSspin^+^ using these models. The KRR models using SLATM, aSLATM, and SPA^H^M(a) achieve strong agreement with the reference values (*R*^2^ = 0.94–0.96), whereas ε-SPA^H^M performs substantially worse (*R*^2^ = 0.40).

For the approach (ii), all KRR models are evaluated (Table S14 in the SI). Across all representations, the Δ*E*_HS–LS_ > 0 subset is more difficult to predict than the Δ*E*_HS–LS_ < 0 subset, consistent with the previous observation in Fig. S4. This is linked to the underlying distribution of the target property within the TM-GSspin^+^. Specifically, the property distribution shows that complexes with Δ*E*_HS–LS_ < 0 are densely concentrated into a narrow, sharp peak, providing the models with highly clustered data points. In contrast, the data points for Δ*E*_HS–LS_ > 0 are scattered across a much broader and flatter energy range with a long tail, making it inherently more difficult for the ML models to accurately learn and generalize in that region.

Another notable observation is that ε-SPA^H^M improves dramatically in Table S14 relative to Table S13, although it still performs substantially worse than the other KRR models. In Table S13, performance is evaluated on the full TM-GSspin^+^, so each reported MAE reflects a mixture of complexes with Δ*E*_HS–LS_ < 0 and Δ*E*_HS–LS_ > 0 except for ε-SPA^H^M. The full-dataset MAE of ε-SPA^H^M is ∼33 kcal mol^−1^, whereas in Table S14 it decreases to ∼10 for Δ*E*_HS–LS_ < 0 and ∼19 for Δ*E*_HS–LS_ > 0. This suggests that a large part of its poor performance on the full dataset arises from the difficulty of handling the coexistence of the two regimes, rather than from uniformly poor performance within each subset. Moreover, because ε-SPA^H^M is built from the occupied orbital eigenvalues of a one-electron guess Hamiltonian, it may lack the resolution required to capture the spin-splitting energies across heterogeneous TM complexes with diverse electronic and structural characteristics, such as variations in metal identity, coordination geometry, and the arrangement of donor atoms affecting ligand field strength. It is also notable that, for ε-SPA^H^M, the choice of spin state used to construct the representation has almost no effect within either subset, as the MAEs are nearly identical across all three variants.

### 4.2 Frontier molecular orbital energies

**Fig. 4:**
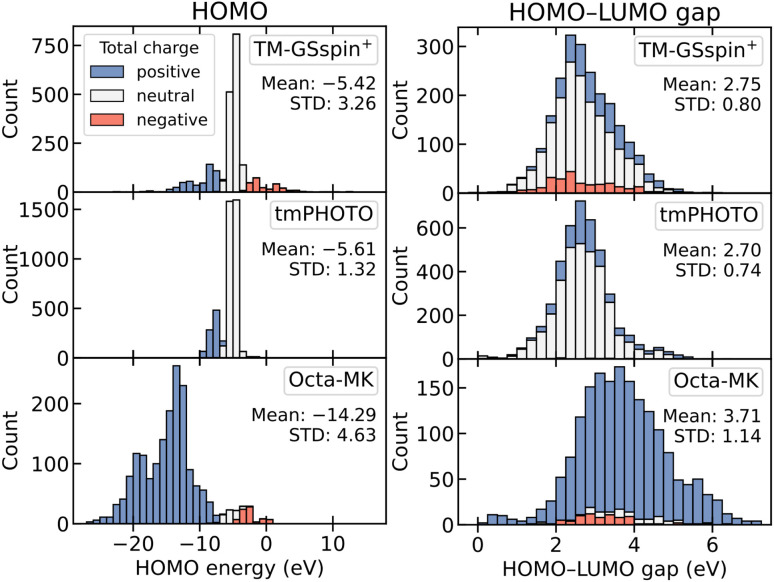
Stacked histograms of HOMO (left) and HOMO–LUMO gap (right) for the benchmark datasets: TM-GSspin^+^ (top), tmPHOTO (middle), and Octa-MK (bottom). Colors represent the total charge of the complexes (blue: positive, white: neutral, red: negative).

For the prediction of frontier molecular orbital energies and their energy gap, all three datasets were used, and only global representations were evaluated, as these are inherently global properties. For open-shell species, the HOMO is defined as the higher energy orbital between the alpha- and beta-spin HOMOs, the LUMO as the lower energy orbital between the alpha- and beta-spin LUMOs, and the HOMO–LUMO gap as the energy difference between these two orbitals. Fig. 4 shows the distributions of HOMO and HOMO–LUMO gap as stacked histograms, where colors indicate the total charge of the complexes (positive, neutral, or negative). The corresponding LUMO distributions are shown in Fig. S6, as they closely resemble those of the HOMO energies.

Comparing the charge distributions across the datasets, TM-GSspin^+^ includes 21.9% positive, 66.9% neutral, and 11.2% negative complexes. tmPHOTO is largely neutral (78.8%), with 20.6% positive and only 0.5% negative charged species. Octa-MK is dominated by positively charged complexes (92.3%), with small fractions of neutral (3.4%) and negative (4.3%) ones. Negatively charged complexes generally exhibit higher (less stabilized) HOMO energies, positively charged complexes show lower (more stabilized) HOMO energies, and neutral species fall between these extremes. As a result, differences in charge composition produce distinct HOMO energy distributions across the datasets. Despite these variations, TM-GSspin^+^ and tmPHOTO share similar mean HOMO energies (approximately −5.5 eV) because both contain large proportions of neutral complexes. Octa-MK instead shows a much lower mean HOMO energy (around −15 eV), reflecting its prevalence of positively charged complexes, which typically feature metal centers in oxidation states II or III coordinated by neutral ligands. The HOMO–LUMO gap, however, shows little dependence on the total charge of the complexes, since changes in charge mainly shift the HOMO and LUMO energies together without significantly affecting the difference between them.

Table 2 shows the MAEs of the models for predicting HOMO, LUMO, and HOMO–LUMO gap, with corresponding standard deviations provided in Table S17. Overall, MACE-QS performs best across all properties. For HOMO and LUMO, incorporating electronic information yields notably lower errors than for the HOMO–LUMO gap, as evidenced by the improvements from 3DMol and MACE to their QS-embedded variants. This effect is most pronounced in TM-GSspin^+^ and Octa-MK, which contain larger fractions of charged species and consequently exhibit broader HOMO and LUMO distributions. In contrast, for the HOMO–LUMO gap, the performance differences between 3DMol/MACE and their charge and spin embedded variants are small or negligible, indicating that electronic information provides limited additional benefit for learning this property.

**Table 2:** Mean absolute errors (MAEs, in eV) for (a) HOMO, (b) LUMO, and (c) HOMO–LUMO gap across datasets. The best-performing models are highlighted in bold

Method	TM-GSspin^+^	tmPHOTO	Octa-MK
**(a) HOMO (eV)**			
SLATM	1.02	0.33	0.74
FCHL	1.36	0.52	1.06
SOAP	1.27	0.44	1.03
ε-SPA^H^M	0.61	0.32	0.40
SPA^H^M(a)	1.50	0.56	1.04
SPA^H^M(b)	0.73	0.32	0.51
3DMol	1.23	0.31	0.87
MACE	1.24	0.30	1.07
3DMol-QS	0.43	**0.18**	0.26
MACE-QS	**0.35**	**0.16**	**0.21**

**(b) LUMO (eV)**			
SLATM	1.07	0.34	0.85
FCHL	1.39	0.55	1.22
SOAP	1.27	0.46	1.17
ε-SPA^H^M	0.74	0.42	0.52
SPA^H^M(a)	1.59	0.60	1.00
SPA^H^M(b)	0.78	0.36	0.55
3DMol	1.30	0.28	1.02
MACE	1.24	0.34	1.23
3DMol-QS	0.50	**0.18**	0.33
MACE-QS	**0.40**	**0.19**	**0.20**

**(c) HOMO–LUMO gap (eV)**			
SLATM	**0.38**	**0.21**	0.45
FCHL	0.43	0.28	0.62
SOAP	**0.40**	0.24	0.55
ε-SPA^H^M	0.54	0.47	0.57
SPA^H^M(a)	0.45	0.32	0.45
SPA^H^M(b)	0.42	0.30	0.43
3DMol	0.43	**0.22**	0.47
MACE	0.43	**0.22**	0.48
3DMol-QS	0.44	**0.22**	0.34
MACE-QS	**0.36**	**0.22**	**0.25**

For the HOMO–LUMO gap, we additionally evaluate a quantum-informed geometric deep learning model developed by Kneiding *et al.*^39^ In their work, the authors introduced the transition metal quantum mechanics graph (tmQMg) dataset, which provides natural quantum graphs (NatQG) for approximately 60 000 TM complexes. NatQG incorporates geometric and electronic information derived from natural bond orbital analysis. We identified 2696 overlapping complexes between tmQMg and tmPHOTO (both are subsets of tmQM^57^), corresponding to about 65% of tmPHOTO. Instead of generating NatQG representations for the remaining complexes, we train only on the overlapping tmPHOTO subset using the NatQG representations and model architectures provided in the authors' GitHub repository,^97^ while following the original training protocol and optimized hyperparameters.

Table S18 presents the MAEs for the tmPHOTO subset obtained using two GNN models based on two types of NatQG graphs. For comparison, we also include the MAEs reported for the corresponding models on the tmQMg test set in the original study, where the lowest MAE for HOMO–LUMO gap prediction was 6.02 mHa (0.164 eV). On the tmPHOTO subset, the best model achieved an MAE of 6.72 mHa (0.183 eV) for the HOMO–LUMO gap, slightly outperforming the best-performing models evaluated on the full tmPHOTO dataset in this work (0.21 to 0.22 eV). This result indicates that NatQG remains highly effective for predicting the HOMO–LUMO gap even in a reduced-data regime.

Comparing the KRR models, ε-SPA^H^M achieves the highest accuracy in predicting HOMO for TM-GSspin^+^ and Octa-MK, followed closely by SPA^H^M(b). This outcome is expected because ε-SPA^H^M is constructed from the occupied orbital energies of a one-electron Hamiltonian and therefore aligns well with the DFT-computed HOMO energies. A similar pattern appears for LUMO prediction: ε-SPA^H^M remains effective, though its MAEs increase by about 0.1 eV relative to HOMO, and SPA^H^M(b) attains nearly comparable accuracy. In tmPHOTO, however, ε-SPA^H^M, SPA^H^M(b), and SLATM reach similar accuracy for HOMO prediction, while SPA^H^M(b) and SLATM perform best for LUMO. These results indicate that although ε-SPA^H^M is a good representation for predicting frontier orbital energies, its advantage diminishes in tmPHOTO, showing that representation performance depends not only on the representation itself but also on dataset characteristics.

For HOMO–LUMO gap prediction, purely structure-based representations perform best. SLATM and SOAP give the lowest MAEs for TM-GSspin^+^, and SLATM remains the strongest performer for tmPHOTO. In Octa-MK, SLATM, SPA^H^M(a) and SPA^H^M(b) achieve similar accuracy. Notably, ε-SPA^H^M performs worst for the gap, despite its superior accuracy for HOMO prediction. SLATM instead emerges as the most robust representation for gap prediction across datasets, even though its individual HOMO and LUMO predictions are less accurate in TM-GSspin^+^ and Octa-MK.

**Fig. 5:**
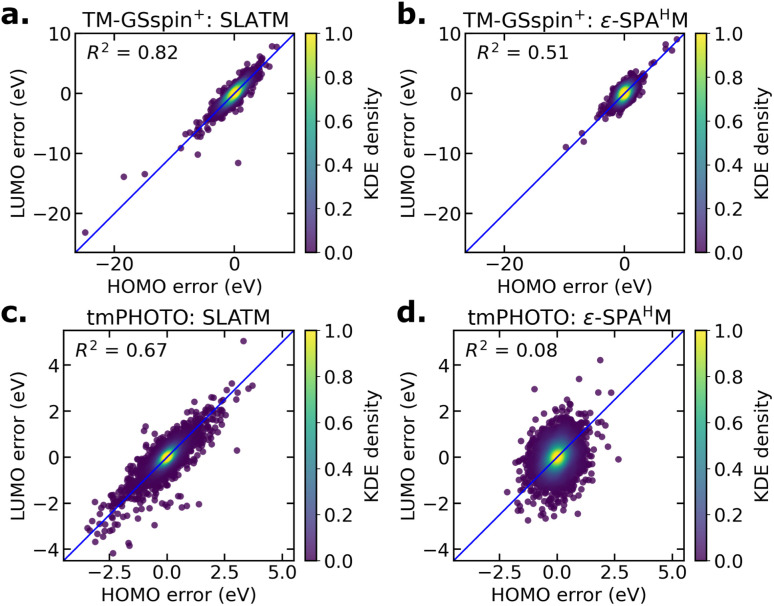
HOMO–LUMO prediction error correlation plots for (a) SLATM and (b) ε-SPA^H^M on TM-GSspin^+^, and (c) SLATM and (d) ε-SPA^H^M on tmPHOTO.

To examine this behavior, we analyze the correlation between HOMO and LUMO prediction errors for SLATM and ε-SPA^H^M, obtained from the 10-fold CV test sets (Fig. 5). In TM-GSspin^+^, SLATM shows strong error cancellation: its larger individual HOMO and LUMO errors are tightly correlated (*R*^2^ = 0.82), which yields much smaller HOMO–LUMO gap errors. ε-SPA^H^M, in contrast, has narrower error distributions but a weaker correlation (*R*^2^ = 0.51), leading to less cancellation and thus larger gap errors. tmPHOTO shows the same overall trend, though the correlation between HOMO and LUMO errors is generally weaker than in TM-GSspin^+^. ε-SPA^H^M exhibits no correlation between HOMO and LUMO errors. SLATM displays much smaller absolute HOMO and LUMO errors in tmPHOTO compared to TM-GSspin^+^, which explains its improved MAEs for frontier orbital energy predictions in tmPHOTO.

**Fig. 6:**
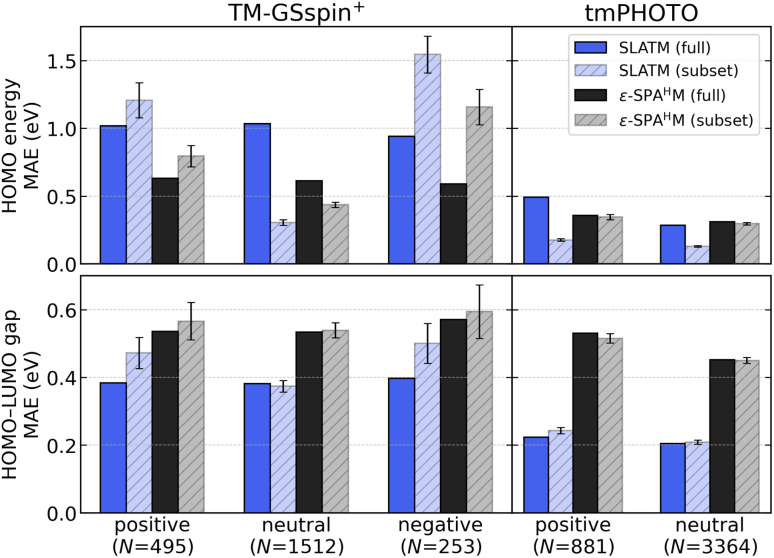
Mean absolute errors (MAEs, in eV) of KRR models using SLATM and ε-SPA^H^M for subsets grouped by molecular charge (positive, neutral, negative). Sample sizes *N* appear below each subset. The plots report HOMO (top) and HOMO–LUMO gap (bottom) MAEs for TM-GSspin^+^ (left) and tmPHOTO (right). Solid bars show subset MAEs from 10-fold CV on the full dataset, and hatched bars show subset MAEs from 5-fold CV on each subset.

We further analyze model performance for HOMO and HOMO–LUMO gap prediction across subsets defined by total molecular charge. MAEs are computed using two approaches: (i) 10-fold CV on the full dataset, followed by grouping the resulting test set errors by charge, and (ii) independent 5-fold CV within each charged subset. Fig. 6 reports the resulting MAEs of KRR models using SLATM and ε-SPA^H^M for each charged subset (positive, neutral, or negative) in TM-GSspin^+^ and tmPHOTO.

In HOMO prediction for TM-GSspin^+^ (upper left in Fig. 6), subset MAEs derived from full-dataset training show that SLATM produces consistently larger errors than ε-SPA^H^M, with both models exhibiting little variation across subsets. When models are trained within each subset, however, their behaviors diverge. Within the neutral subset, both improve and SLATM even outperforms ε-SPA^H^M, indicating that a structure-only representation is effective when the dataset is restricted to neutral species. On the positive and negative subsets, MAEs increase for both models, but ε-SPA^H^M maintains lower errors, reflecting its superior ability to handle charged species.

The negative subset of tmPHOTO is omitted due to its small size. In HOMO prediction for tmPHOTO (upper right in Fig. 6), full-dataset training yields higher subset MAEs for SLATM than for ε-SPA^H^M in the positive subset and similar MAEs in the neutral subset, indicating that SLATM handles charged systems less effectively when mixed charge states are present. Training within each subset lowers SLATM errors and produces similar MAEs for the positive and neutral subsets, lowing SLATM to outperform ε-SPA^H^M. In contrast, the MAEs of ε-SPA^H^M remain nearly unchanged across the two evaluation approaches.

These trends are explained by the HOMO distributions for each charge subset (Fig. S7, which provides additional detail). Neutral subsets in both datasets are symmetric, whereas the positive and negative subsets in TM-GSspin^+^ are strongly skewed, yielding larger subset MAEs when models are trained only on charged species because kernel methods perform poorly in sparse-tail regions. The overall TM-GSspin^+^ distribution is less skewed, which allows better generalization across charge when the full dataset is used. In tmPHOTO, the positive and neutral subsets exhibit nearly symmetric HOMO distributions, which reduces the learning difficulty for models trained within these subsets.

In HOMO–LUMO gap prediction for TM-GSspin^+^ (bottom left in Fig. 6), SLATM achieves lower MAEs than ε-SPA^H^M for every charge subset. The neutral subset shows almost no difference between full-dataset and within-subset evaluations for either model. For the positive and negative subsets, training within each subset leads to only a slight increase in MAEs relative to the full-dataset results, and these changes remain small for SLATM and essentially negligible for ε-SPA^H^M.

A similar pattern appears in tmPHOTO (bottom right in Fig. 6). SLATM again provides lower MAEs for both the positive and neutral subsets, and the two evaluation strategies yield nearly identical results for each model. Together, these results show that SLATM maintains a consistent advantage over ε-SPA^H^M for predicting the HOMO–LUMO gap, independent of molecular charge, while the task itself is largely unaffected by the choice of evaluation protocol or dataset composition. The corresponding gap distributions for each charge subset appear in Fig. S8.

In summary, when a dataset contains electronically diverse charged species, as in TM-GSspin^+^, and the target-property distribution depends strongly on the total molecular charge, as in the HOMO energies, ε-SPA^H^M achieves higher accuracy because it encodes electronic information that SLATM, a purely structure-based representation, does not capture. This pattern is also reflected in the neutral subset: with full-dataset training, ε-SPA^H^M performs better, but when training is restricted to neutral species, SLATM attains lower errors. For the HOMO–LUMO gap, whose distribution shows little dependence on total charge, SLATM achieves consistently lower errors than ε-SPA^H^M across datasets and charge subsets. These results show that when a dataset spans a wide range of charge and spin states and the target-property distribution varies strongly with electronic character, model performance depends critically on whether the model reflects electronic information. The dataset composition also matters; whether it is dominated by neutral species or balanced across charge states determines the extent to which electronically informed models offer a clear advantage over structure-only models. Additionally, learning curves for the KRR models used to predict frontier orbital energies and energy gaps are provided in Fig. S9.

### 4.3 Dipole moment

**Fig. 7:**
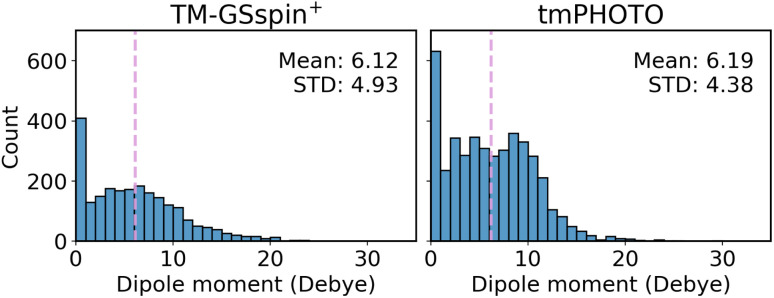
Distribution of dipole moment magnitudes in TM-GSspin^+^ (left) and tmPHOTO (right). The dashed line denotes the mean value.

For dipole moment prediction, global representations are evaluated in the same manner as for HOMO, LUMO, and their energy gap. The distributions of dipole moment magnitudes for TM-GSspin^+^ and tmPHOTO are shown in Fig. 7. Their mean and standard deviation are similar, and the distributions do not exhibit any dependence on the total charge or spin of the complexes (Fig. S10).

Table 3 summarizes the MAEs for dipole moment magnitudes, with standard deviations provided in Table S19. All models show lower errors on tmPHOTO than on TM-GSspin^+^, reflecting its larger dataset size despite their similar property distributions. MACE with the modified AtomicDipolesMACE architecture achieves the best performance by predicting the full dipole vector and then computing its magnitude, outperforming all models that predict only a scalar value across both datasets. The superior performance of AtomicDipolesMACE relative to scalar-predicting models likely stems from its equivariant architecture, which explicitly preserves rotational symmetry and captures directional information in local atomic environments, both of which are essential for accurately modeling vector properties such as dipole moments. This allows the model to learn the spatial distribution and orientation of charges more effectively than scalar-predicting models that regress only the dipole moment magnitude. The dipole moment magnitude is then obtained from the predicted vector, which may lead to improved generalization compared to directly learning a scalar target. Additionally, using the same procedure as for the HOMO–LUMO gap, the NatQG-based GNN model evaluated on the tmPHOTO subset (Table S18) achieves an MAE of 1.645 debye for dipole moment magnitude, comparable to those for tmPHOTO obtained with 3DMol and 3DMol-QS (about 1.60 debye).

**Table 3:** Mean absolute errors (in Debye) for predicting dipole moment magnitudes for TM-GSspin^+^ and tmPHOTO. MACE employs a modified AtomicDipolesMACE equivariant architecture. The best-performing models for each dataset are highlighted in bold

Method	TM-GSspin^+^	tmPHOTO
SLATM	2.42	1.53
FCHL	2.44	1.81
SOAP	2.20	1.60
ε-SPA^H^M	3.45	2.83
SPA^H^M(a)	3.06	2.19
SPA^H^M(b)	2.83	2.00
3DMol	1.97	1.60
MACE (AtomicDipolesMACE, equi.)	**1.60**	**1.05**
3DMol-QS	2.13	1.62

Among KRR models, those using purely structure-based representations consistently perform better than the quantum-informed SPA^H^M family, indicating that electronic information is less critical for dipole moment prediction than for frontier orbital energies or spin-splitting energies. We also evaluate MACE architectures originally developed for energy prediction (Table S20). These energy-targeted models perform poorly for dipole magnitudes, and their accuracy further degrades when charge, spin, or both embeddings are added. This demonstrates that accurate dipole moment prediction requires architectures designed for vector quantities, even when the final target is scalar.

### 4.4 Timings and representation sizes

Lastly, we assess the computational efficiency of the models to provide a comprehensive comparison across methods. To ensure a fair assessment, we use subsets of 500 randomly selected complexes from the TM-GSspin^+^ and tmPHOTO. For the KRR models, we measure both representation generation and kernel computation times. Table S21 reports the corresponding wall times, averaged over five independent subsets, together with representation sizes obtained from the full datasets. All timings are obtained on a single CPU core of an Intel Xeon Gold 5220R node (48 cores, 2.20 GHz).

SOAP is the fastest global representation to generate, requiring less than one minute, whereas SLATM takes about twenty five minutes for the same TM-GSspin^+^ subset. Both representations remain inefficient in Laplacian kernel construction because their sizes are large. The most compact representation is ε-SPA^H^M, which allows kernel computation in 0.1 seconds, but requires approximately twenty minutes to generate, making it the second slowest. When considering the total time for representation generation and kernel computation, SOAP is the most efficient global representation overall.

For the local representations, we evaluate aSLATM, SOAP, SPA^H^M(a), and SPA^H^M(b), measuring the time required to generate representations for the metal centers and to compute the kernels. Local FCHL is excluded because its implementation generates representations for all atoms before extracting that of the metal center, which results in timings similar to the global variant. To ensure a fair comparison, we modify the 
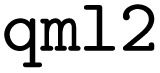
 code^91^ so that aSLATM generates atomic representations only for metal elements. Local SOAP and aSLATM are faster to generate than their global counterparts, while their kernel computation times remain similar because the representation sizes do not change. SPA^H^M(a) and SPA^H^M(b) require substantially longer generation times, which leads to much higher computational cost and limits their efficiency.

In tmPHOTO, the presence of 25 distinct elements (compared to 18 in TM-GSspin^+^) increases the size of the representations and lengthens both representation generation and kernel computation. Although the absolute timings differ, the overall behavior is unchanged. Representation generation is the dominant cost, and SOAP remains the most efficient option on a single CPU for both global and local representations. ε-SPA^H^M is the most compact and therefore enables rapid kernel construction, but its long generation time remains a major limitation. This drawback becomes even more pronounced in SPA^H^M(a) and SPA^H^M(b), whose high generation cost restricts their practical usefulness. Using the Gaussian kernel instead of the Laplacian kernel greatly reduces kernel computation, particularly benefiting SLATM and SOAP once the representations are prepared (Table S22).

Finally, in Table 4, we estimate training and test times of the KRR models from two measured quantities: the representation generation time *R* (measured for 500 molecules) and the kernel construction time *K* (measured for forming a 500 × 500 kernel matrix). Consistent with the 90/10 train/test split used in 10-fold CV, we estimate the training cost as 0.9*R* for generating representations for the training set plus 0.81*K* for constructing the training–training kernel (0.9 × 0.9), yielding a total training time of 0.9*R* + 0.81*K*; the additional matrix-inversion cost is negligible at this scale. Likewise, we estimate the test cost as 0.1*R* for generating representations for the test set plus 0.09*K* for evaluating the training-test kernel (0.9 × 0.1), yielding a total test time of 0.1*R* + 0.09*K*; associated linear-algebra costs are also negligible. This provides a simple and transparent estimate of both training and test times for KRR models under our CV protocol.

**Table 4:** Estimated times in seconds for KRR models on subsets of TM-GSspin^+^ and tmPHOTO, each containing 500 randomly selected complexes. Training (“train”) and testing (“test”) times are estimated for a 90/10 train/test split from the measured representation generation and Laplacian kernel construction times for the same subsets (see Table S21). The “repr. size” column denotes the size of the respective representations (the number of features). All values are averaged over five subsets for each dataset

Method	Subset of TM-GSspin^+^			Subset of tmPHOTO		
	Train	Test	repr. size	Train	Test	repr. size
**Global**						
SLATM	1416	157	398 321	5728	636	1 009 514
FCHL	885	98	983	1039	115	13 600
SOAP	59	7	103 680	127	14	200 000
ε-SPA^H^M	1068	119	736	2525	281	902

**Local**						
aSLATM	858	95	398 321	4034	448	1 009 514
SOAP	26	3	103 680	49	5	200 000
SPA^H^M(a)	30 783	3420	15 342	69 564	7729	19 980
SPA^H^M(b)	18 084	2009	9972	39 329	4370	13 850

Timings for the geometric deep learning models are obtained on a single NVIDIA L40s GPU node. For each model, we record the time required for initialization, 128 training epochs, and evaluation on the test set for HOMO–LUMO gap prediction, as summarized in Table 5. The results show that 3DMol is markedly faster than both the KRR methods and MACE, offering the highest computational efficiency among all models evaluated. Incorporating charge or spin embeddings in MACE adds only a few minutes to the runtime and does not meaningfully affect efficiency. Invariant and equivariant MACE are also compared, and the equivariant MACE requires roughly three to four times more computation for the same subsets. Finally, we extend the timing analysis of 3DMol to the full benchmark datasets using the same procedure (Table S23). The model continues to run efficiently, completing the entire workflow within a few minutes. Since computational cost naturally increases with dataset size, this behavior highlights the strong efficiency of 3DMol and its suitability for handling very large prediction tasks.

**Table 5:** Elapsed times in seconds for 3DMol and MACE on subsets of TM-GSspin^+^ and tmPHOTO, each consisting of 500 randomly chosen complexes. Reported times are for initialization (“init”), training for 128 epochs (“train”), and test-set evaluation (“test”) for HOMO–LUMO gap prediction. The “repr. size” column lists the dimensionality of the learned representation. Values for the subsets are averaged over five subsets for each dataset. Both invariant (in.) and equivariant (equi.) MACE models are included

Method	Subset of TM-GSspin^+^				Subset of tmPHOTO			
	Init	Train	Test	repr. size	Init	Train	Test	repr. size
3DMol	7.8	41	0.1	64	12	42	0.1	64
MACE (in.)	1.6	3978	0.9	512	1.7	5099	1.3	512
MACE (equi.)	2.1	15 313	4.0	2560	2.2	20 371	5.6	2560

## 5 Conclusions

In this work, we present a systematic benchmark of physics-inspired ML models for predicting quantum-chemical properties of mononuclear TM complexes using three complementary datasets. The models include KRR models based on molecular representations and geometrical deep learning models.

Across all datasets, models that incorporate electronic information, either implicitly through quantum-mechanical operators or explicitly through charge and spin embeddings, consistently outperform purely structure-based models for predicting spin-splitting energies, HOMO and LUMO energies, whose property distributions are strongly governed by spin or charge states. For energy-related properties, MACE-QS is the best overall performer. For dipole moment magnitudes, AtomicDipolesMACE, which predicts the full dipole vector before computing its magnitude, substantially surpasses models that directly predict a scalar value. Among molecular representations, ε-SPA^H^M performs well for frontier orbital energies predictions when the dataset includes diverse charged species, although it performs poorly for their energy gap. In contrast, SLATM shows the opposite trend and remains robust for HOMO–LUMO gap prediction across datasets, likely due to strong error cancellation between HOMO and LUMO prediction errors.

The results highlight how dataset composition, the diversity of charge and spin states, and the target property distributions shaped by these electronic characteristics influence the relative performance of purely structure-based and quantum-informed ML models. We note, however, that while the observed trends are consistent and informative for practitioners, the quantitative outcomes may be sensitive to the specific data-generation pipeline. When the target property distribution is relatively insensitive to electronic characteristics, structure-only models outperform quantum-informed models, since capturing geometric differences becomes more critical for achieving accurate predictions. When the property distribution strongly depends on electronic characteristics, models incorporating electronic information provide clear advantages. This advantage becomes more pronounced when the dataset contains a balanced mixture of charge states rather than being dominated by neutral species. In this context, geometric deep learning models with additional charge and spin embeddings (MACE-QS and 3DMol-QS) achieve high accuracy across datasets and target properties. Timing analysis further shows that 3DMol provides high computational efficiency, making it well suited for large-scale prediction tasks.

In summary, this study provides insight into when geometric information alone is sufficient and when electronic information becomes essential for physics-based ML models applied to TM complexes. These insights help researchers select effective models by considering the electronic characteristics and diversity of the dataset, the target property, and available computational resources. These findings also help guide the further development of physics-inspired ML models capable of handling datasets with varied charge and spin states.

## Author contributions

Y. C. and C. C. conceived the project. Y. C. curated the dataset, generated the molecular representations, and performed the training and evaluation of the KRR and MACE models. K. R. B. carried out the training and evaluation of the 3DMol models and measured their runtimes. Y. C. A. trained NatQG models and aided Y. C. in generating the SPA^H^M representations and measuring the timings for the KRR models. All authors discussed the results. The original manuscript was written by Y. C. with help and feedback from all authors. C. C. provided supervision throughout and is acknowledged for funding acquisition.

## Conflicts of interest

There are no conflicts to declare.

## Supplementary Material

DD-005-D5DD00571J-s001

## Data Availability

All data and code used in this work are available at https://github.com/lcmd-epfl/benchmark_tmc. Although the original datasets were published as open-source resources, we applied several filtering steps and modifications. Therefore, the final versions of three datasets used in this work are provided in the GitHub repository and in the Materials Cloud at https://doi.org/10.24435/materialscloud:pv-nj. The GitHub repository provides scripts for generating molecular representations, measuring computational timings, and performing 10-fold cross-validation with 
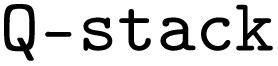
, and running the 3DMol and MACE models. A detailed explanation of the workflow and file structure is provided in the README. The Materials Cloud repository additionally contains all molecular representations and the trained geometric deep learning models. Supplementary information (SI) is available. See DOI: https://doi.org/10.1039/d5dd00571j.
