# Supplementary material for: Benchmarking physics-inspired machine learning models for transition metal complexes with diverse charge and spin states
Source: Digit Discov. 2026 Apr 28;5(5):2103–19. doi: 10.1039/d5dd00571j (PMC13150962; doi:10.1039/d5dd00571j)
Supplement: DD-005-D5DD00571J-s001 [file DD-005-D5DD00571J-s001.pdf]

Supplementary Information (SI) for Digital Discovery.
This journal is © The Royal Society of Chemistry 2026

Supplementary Information: Benchmarking physics-inspired machine learning models for transition metal complexes with diverse charge and spin states

Yuri Cho,[†,‡] Ksenia R. Briling,[†] Yannick Calvino Alonso,[†,‡] Ruben Laplaza,[†,¶] and Clemence Corminboeuf[*,†,‡,¶]

†*Laboratory for Computational Molecular Design, Institute of Chemical Sciences and Engineering, École Polytechnique Fédérale de Lausanne (EPFL), Lausanne, Switzerland*

‡*National Centre for Computational Design and Discovery of Novel Materials (MARVEL), École Polytechnique Fédérale de Lausanne (EPFL), Lausanne, Switzerland*

¶*National Centre for Competence in Research–Catalysis (NCCR–Catalysis), École Polytechnique Fédérale de Lausanne (EPFL), Lausanne, Switzerland*

E-mail: clemence.corminboeuf@epfl.ch

S1 Benchmark datasets

S1.1 Correction applied in TM-GSspin$^+$

The complexes in TM-GSspin$^+$ were originally obtained from a database[1] we previously extracted from the CSD using `cell2mol` v1.1.0, where the assigned metal oxidation states agreed with those reported in the crystallographic information file (CIF). During the dataset validation process, we identified a few complexes showing discrepancies between the net charges assigned by `cell2mol` and those reported in the corresponding CIF, primarily due to errors in the adjacency matrix generated by `cell2mol` v1.1.0. In this earlier version, smaller covalent radii were applied to alkali and alkaline earth metals to avoid conflicts in SMILES generation with RDKit. As a result, transition metal complexes containing these elements were often incorrectly treated as disconnected. Additionally, a few complexes included solvent molecules or counterions that were mistakenly recognized as ligands when their interatomic distances were close to the bonding threshold.

We corrected these entries to ensure consistency in both structure and net charge with the moieties reported in the CIFs, and subsequently performed the necessary computations. It is important to note that these adjustments did not alter the metal oxidation states or the ground state spin determined in our previous works.[1,2]

S1.2 Exclusion of complexes with rarely occurring elements

A larger variety of element types in a dataset increases the size of the molecular representation. To avoid excessive sparsity, we excluded complexes containing rarely occurring elements. Table S1 summarizes the element composition of each dataset and the number of complexes removed due to the presence of elements appearing in fewer than 1% of the original dataset.

Table S1: Element composition and the number of excluded complexes due to the presence of rarely occurring elements for each dataset.

	TM-GSspin^{+}	tmPHOTO	Octa-MK
List of element types	H, B, C, N, O, F, Si, P, S, Cl, Cr, Mn, Fe, Co, Ni, Se, Br, I	H, B, C, N, O, F, Si, P, S, Cl, Fe, Ni, Cu, Zn, Br, Ru, Pd, Ag, Cd, I, Re, Ir, Pt, Au, Hg	H, C, N, O, F, P, S, Cl, I, Cr, Mn, Fe, Co
List of rarely occurring elements	Li, Na, Mg, Al, K, Ga, As, Sn, Sb, Te, Bi	Sc, Ti, V, Cr, Mn, Co, As, Se, Y, Zr, Mo, Rh, Hf, Ta, W, Os	–
# excluded complexes	71	232	0

S2 Machine learning models

S2.1 cMBDF, MODA, and MAOC

Table S2 summarizes the performances of cMBDF, MODA, and PC3-MAOC, which were evaluated across the benchmark datasets but are not discussed in the main text.

cMBDF[3,4] is a highly compact atomic representation that encodes the local chemical environment through translationally and rotationally invariant functionals of smooth atom-centered Gaussian density distributions weighted by many–body interaction potentials.

MODA[5] is constructed from the density matrix, obtained using the Superposition of Atomic Densities (SAD) initial guess,[6] for a subset of molecular orbitals and can be adapted to decouple intra- and intermolecular interactions. In this work, we use MODA with intramolecular features, since we are dealing with single molecules.

MAOC[7] is constructed from the coefficients of localized guess molecular orbitals, which are linear combinations of predefined orthogonalized atomic orbitals. In this work, we employ a reduced-dimensionality version, PCX-MAOC, obtained through principal component analysis, specifically three principal components of PC3-MAOC.

Table S2: Performances of cMBDF, MODA, and PC3-MAOC across datasets. MAE: mean absolute error, STD: standard deviation. For MODA and PC3-MAOC, different basis sets were used to construct the representations, as indicated in parentheses.

(a) Spin-splitting energy (kcal/mol)

	TM-GSspin$^+$		Octa-MK	
method	MAE	STD	MAE	STD
cMBDF	12.44	0.88	5.50	0.37
MODA (3-21G)	22.24	1.55	9.72	0.61
MODA (def2-TZVP)	21.55	1.02	9.56	0.63
PC3-MAOC (3-21G)	36.04	2.04	16.31	0.57
PC3-MAOC (def2-SVP)	37.91	2.04	18.23	0.90
PC3-MAOC (def2-TZVP)	39.58	1.60	16.12	0.69

(b) HOMO (eV)

	TM-GSspin$^+$		tmPHOTO		Octa-MK	
method	MAE	STD	MAE	STD	MAE	STD
cMBDF	1.22	0.08	0.46	0.02	0.94	0.07
MODA (3-21G)	1.63	0.09	–	–	2.22	0.13
MODA (def2-TZVP)	1.65	0.10	0.49	0.03	2.21	0.11
PC3-MAOC (3-21G)	1.96	0.17	–	–	2.31	0.16
PC3-MAOC (def2-SVP)	1.98	0.16	0.96	0.03	2.26	0.18
PC3-MAOC (def2-TZVP)	1.99	0.16	0.96	0.03	2.55	0.17

(c) LUMO (eV)

	TM-GSspin$^+$		tmPHOTO		Octa-MK	
method	MAE	STD	MAE	STD	MAE	STD
cMBDF	1.24	0.06	0.48	0.02	1.11	0.10
MODA (3-21G)	1.61	0.09	–	–	2.57	0.13
MODA (def2-TZVP)	1.61	0.10	0.52	0.03	2.63	0.12
PC3-MAOC (3-21G)	1.94	0.18	–	–	2.62	0.14
PC3-MAOC (def2-SVP)	1.97	0.16	1.01	0.02	2.54	0.18
PC3-MAOC (def2-TZVP)	1.99	0.16	1.00	0.03	2.74	0.17

(d) HOMO–LUMO gap (eV)

	TM-GSspin$^+$		tmPHOTO		Octa-MK	
method	MAE	STD	MAE	STD	MAE	STD
cMBDF	0.42	0.02	0.25	0.01	0.51	0.04
MODA (3-21G)	0.50	0.03	–	–	0.75	0.03
MODA (def2-TZVP)	0.50	0.03	0.29	0.01	0.76	0.02
PC3-MAOC (3-21G)	0.57	0.02	–	–	0.86	0.02
PC3-MAOC (def2-SVP)	0.58	0.02	0.52	0.02	0.85	0.03
PC3-MAOC (def2-TZVP)	0.59	0.03	0.52	0.02	0.85	0.02

(e) Dipole moment (Debye)

	TM-GSspin$^+$		tmPHOTO	
method	MAE	STD	MAE	STD
cMBDF	2.39	0.05	1.70	0.11
MODA (3-21G)	3.00	0.13	–	–
MODA (def2-TZVP)	2.93	0.16	1.90	0.10
PC3-MAOC (3-21G)	3.68	0.13	–	–
PC3-MAOC (def2-SVP)	3.75	0.12	3.20	0.13
PC3-MAOC (def2-TZVP)	3.76	0.19	3.19	0.10

S2.2 Equivariant and invariant MACE

Table S3: Comparison of equivariant (equi.) and invariant (in.) MACE and MACE-QS models. MAE: mean absolute error, STD: standard deviation.

(a) Spin-splitting energy (kcal/mol)

	TM-GSspin$^+$		Octa-MK	
method	MAE	STD	MAE	STD
MACE (equi.)	10.15	0.75	4.81	0.50
MACE (in.)	10.26	1.08	5.18	0.45
MACE-QS (equi.)	8.37	0.49	2.98	0.15
MACE-QS (in.)	8.43	0.61	3.01	0.12

(b) HOMO (eV)

	TM-GSspin$^+$		tmPHOTO		Octa-MK	
method	MAE	STD	MAE	STD	MAE	STD
MACE (equi.)	1.19	0.09	0.30	0.04	1.01	0.06
MACE (in.)	1.24	0.11	0.30	0.04	1.07	0.11
MACE-QS (equi.)	0.34	0.04	0.14	0.01	0.21	0.02
MACE-QS (in.)	0.35	0.04	0.16	0.01	0.21	0.01

(c) LUMO (eV)

	TM-GSspin$^+$		tmPHOTO		Octa-MK	
method	MAE	STD	MAE	STD	MAE	STD
MACE (equi.)	1.21	0.09	0.33	0.03	1.21	0.09
MACE (in.)	1.24	0.09	0.34	0.06	1.23	0.07
MACE-QS (equi.)	0.40	0.06	0.18	0.01	0.20	0.02
MACE-QS (in.)	0.40	0.06	0.19	0.01	0.20	0.03

(d) HOMO–LUMO gap (eV)

	TM-GSspin$^+$		tmPHOTO		Octa-MK	
method	MAE	STD	MAE	STD	MAE	STD
MACE (equi.)	0.41	0.04	0.22	0.02	0.46	0.03
MACE (in.)	0.43	0.04	0.22	0.01	0.48	0.02
MACE-QS (equi.)	0.35	0.03	0.22	0.01	0.24	0.01
MACE-QS (in.)	0.36	0.03	0.22	0.01	0.25	0.02

S2.3 Molecular representations parameters

Table S4: Parameters for SLATM.

Parameter	Value
Gaussian width (2- and 3-body parts)	0.05
grid spacing for distances and angles (2- and 3-body parts)	0.03
cut-off radius	4.8 Å
power in the 2-body potential	6 (London potential)

Table S5: Parameters for FCHL19.

Parameter	Value
number of bins (pair or triplets) of element types in 2-body spectra	24
number of bins (pair or triplets) of element types in 3-body spectra	20
width of the radial distribution function (2-body)	0.32 $Å^2$
width of the radial distribution function (3-body)	2.7 $Å^{-2}$
decay of the 2-body scaling function	1.8
decay of the 3-body scaling function	0.57
weight factor of the 3-body part	13.4
width of Gaussian function used in the Fourier series	π
cut-off radius	8.0 Å

Table S6: Parameters for SOAP. These parameters were taken from Lopanitsyna *et al.*,[8] which used SOAP features to build a potential capable of describing 25 transition metals.

Parameter	Value
cutoff radius	5.0 Å
cutoff smoothing type	ShiftedCosine
cutoff smoothing width	0.5
density type	Gaussian
density width	0.3
basis type	TensorProduct
basis max angular	4
basis radial type	Gto
basis max radial	8
basis spline accuracy	10^{-6}

Table S7: Parameters for the SPA^{H}M representations.

Parameter	ε-SPA^{H}M	SPA^{H}M(a)	SPA^{H}M(b)
guess Hamiltonian	LB	LB	LB
basis-set	minao	minao	minao
effective core potential	cc-pVDZ-pp	cc-pVDZ-pp	cc-pVDZ-pp
auxiliary basis-set	def2-TZVP-JKFIT	–	–
radial cutoff	–	–	10^5 Å

S2.4 3DMol hyperparameters

Table S8 shows the search space for 3DMol hyperparameters, which are explained in details in Ref. 9. The results of the search are presented in Tables S9, S10 and S11.

Table S8: Search space for 3DMol hyperparameters.

Parameter	Grid
Adam initial learning rate	$[5 \cdot 10^{-5}, 10^{-4}, 5 \cdot 10^{-4}, 10^{-3}]$
Adam weight decay parameter	$[10^{-5}, 10^{-4}, 10^{-3}, 0]$
node and edge features embedding size (n_s)	[16, 32, 48, 64]
number of edge features (n_g)	[16, 32, 48, 64]
number of convolutional layers (n_{conv})	[2, 3]
radial cutoff (r_{max})	[2.5, 5.0, 10.0]
maximum number of atom neighbors (n_{neigh})	[10, 25, 50]
dropout probability (p_d)	[0.0, 0.05, 0.1]
`sum_mode`	[`node`, `both`]
`graph_mode`[a]	[`energy`, `vector`]

[a]For the global variant

Table S9: Best 3DMol hyperparameters for TM-GSspin$^+$ in the global and local variants.

Parameter	Dipole moment	HOMO	LUMO	Gap	Spin-splitting E
Global					
n_s	64	64	64	64	64
n_g	16	16	32	32	16
n_{conv}	3	3	3	3	3
r_{max}, Å	10	2.5	2.5	2.5	5
n_{neigh}	10	50	25	50	50
p_d	0.05	0.05	0.1	0	0
learning rate	10^{-3}	10^{-3}	$5 \cdot 10^{-4}$	10^{-3}	10^{-3}
weight decay	10^{-5}	10^{-5}	10^{-3}	10^{-5}	10^{-5}
`sum_mode`	`node`	`node`	`both`	`both`	`both`
`graph_mode`	`energy`	`energy`	`energy`	`vector`	`energy`
Local					
n_s	64	64	48	64	64
n_g	16	32	64	48	64
n_{conv}	3	3	3	3	2
r_{max}, Å	10	5	5	2.5	5
n_{neigh}	10	50	50	50	25
p_d	0.1	0.1	0	0.1	0.05
learning rate	$5 \cdot 10^{-4}$	10^{-3}	$5 \cdot 10^{-4}$	10^{-3}	10^{-3}
weight decay	10^{-5}	10^{-5}	0	10^{-5}	10^{-5}
`sum_mode`	`node`	`node`	`node`	`both`	`node`

Table S10: Best 3DMol hyperparameters for tmPHOTO in the global and local variants.

Parameter	Dipole moment	HOMO	LUMO	Gap
Global				
n_s	64	64	64	64
n_g	64	32	64	64
n_{conv}	3	3	3	3
r_{max}, Å	2.5	2.5	2.5	2.5
n_{neigh}	50	50	50	25
p_d	0.05	0	0.05	0.05
learning rate	10^{-3}	$5 \cdot 10^{-4}$	10^{-3}	10^{-3}
weight decay	10^{-5}	10^{-5}	10^{-5}	10^{-5}
`sum_mode`	`node`	`both`	`node`	`node`
`graph_mode`	`vector`	`vector`	`vector`	`energy`
Local				
n_s	32	64	64	48
n_g	16	64	64	64
n_{conv}	3	3	3	3
r_{max}, Å	5	2.5	2.5	2.5
n_{neigh}	50	25	10	25
p_d	0.1	0.05	0	0.05
learning rate	10^{-3}	$5 \cdot 10^{-4}$	10^{-3}	10^{-3}
weight decay	10^{-5}	10^{-5}	10^{-5}	10^{-5}
`sum_mode`	`both`	`node`	`both`	`node`

Table S11: Best 3DMol hyperparameters for Octa-MK in the global and local variants.

Parameter	HOMO	LUMO	Gap	Spin-splitting E
		Global		
n_s	64	48	64	64
n_g	64	64	32	16
n_{conv}	2	3	2	3
r_{max}, Å	2.5	2.5	2.5	2.5
n_{neigh}	10	25	25	50
p_d	0.05	0.1	0	0
learning rate	$1 \cdot 10^{-3}$	$1 \cdot 10^{-3}$	$1 \cdot 10^{-3}$	$5 \cdot 10^{-4}$
weight decay	$1 \cdot 10^{-5}$	$1 \cdot 10^{-5}$	$1 \cdot 10^{-5}$	$1 \cdot 10^{-5}$
`sum_mode`	node	node	node	both
`graph_mode`	vector	vector	vector	vector
		Local		
n_s	64	64	48	64
n_g	64	64	48	32
n_{conv}	3	3	3	3
r_{max}, Å	5	5	2.5	2.5
n_{neigh}	10	50	10	25
p_d	0	0.05	0.1	0
learning rate	$1 \cdot 10^{-3}$	$1 \cdot 10^{-3}$	$1 \cdot 10^{-3}$	$5 \cdot 10^{-4}$
weight decay	$1 \cdot 10^{-5}$	$1 \cdot 10^{-5}$	$1 \cdot 10^{-5}$	$1 \cdot 10^{-5}$
`sum_mode`	node	node	both	both

S2.5 MACE hyperparameters

Table S12: Hyperparameters of MACE models for energy or dipole moment magnitude prediction. The modified MACE implementation is available in a forked repository at https://github.com/lcmd-epfl/tmc_mace/tree/TMC-benchmark-v1.

Hyperparameter	Energy	Dipole moment magnitude
valid_fraction	0.111112	0.111112
scaling	no_scaling	no_scaling
E0s	average	average
Model Architecture		
model	`MACE`[a]	`AtomicDipolesMACE`[b]
num_interactions	2	2
num_channels	256	256
max_L	2 (equivariant), 0 (invariant)	2 (equivariant)
correlation	3	3
r_max	5.0	5.0
MLP_irreps	128x0e	128x0e
radial_MLP	[128, 256, 512, 1024]	[128, 256, 512, 1024]
default_dtype	float64	float64
Loss and Weights		
energy_weight	10	0
forces_weight	0	0
dipole_weight	–	10
loss	weighted[c]	dipole_magnitude[d]
Training Setup		
batch_size	2	2
valid_batch_size	2	2
max_num_epochs	650	650
start_swa	450	–
eval_interval	1	1
compute_forces	False	False
Optimization		
lr	0.001	0.001
scheduler_patience	5	5
patience	15	15
ema	True	True
ema_decay	0.999	0.999
swa	True	False

[a] Sum pooling replaced by mean pooling for intensive properties prediction
[b] Modified for dipole moment magnitude prediction
[c] Forces excluded from the loss
[d] Weighted mean squared error between reference and predicted dipole magnitudes

For MACE-QS, we additionally include the `--embedding_specs` hyperparameter:

```
--embedding_specs='{
  "total_spin": {"type": "categorical", "per": "graph", "in_dim": 1, "
     emb_dim": 1024, "num_classes": 101, "offset": 0},
  "total_charge": {"type": "categorical", "per": "graph", "in_dim": 1, "
     emb_dim": 1024, "num_classes": 201, "offset": 100}
}'
```

S3 Results

S3.1 Spin-splitting energy

Table S13: Prediction accuracy for spin-splitting energies (in kcal/mol) on TM-GSspin$^+$ and Octa-MK. MAE denotes the mean absolute error, and STD refers to the standard deviation across the folds in 10-fold cross-validation.

	TM-GSspin$^+$		Octa-MK	
method	MAE	STD	MAE	STD
Global				
SLATM	9.33	0.76	4.62	0.33
FCHL	13.11	0.75	6.09	0.35
SOAP	11.47	0.97	5.14	0.29
ε-SPA^{H}M	32.93	1.21	7.69	0.76
SPA^{H}M(a)	10.07	0.48	3.98	0.34
SPA^{H}M(b)	9.25	0.62	4.37	0.34
3DMol	9.27	0.72	4.59	0.32
MACE	10.26	1.08	5.18	0.45
3DMol-QS	8.46	1.18	3.05	0.26
MACE-QS	8.43	0.61	3.01	0.12
Local				
SLATM	8.36	0.71	4.39	0.32
FCHL	12.55	0.48	10.47	0.70
SOAP	8.99	0.59	5.19	0.35
SPA^{H}M(a)	7.58	0.46	3.60	0.24
SPA^{H}M(b)	9.80	0.48	5.55	0.31
3DMol	9.46	0.82	4.50	0.28
3DMol-QS	8.77	0.87	2.92	0.19

For FCHL on Octa-MK dataset, we additionally construct a semi-local FCHL variant by summing the atomic representations of the metal center and its six coordinating atoms. This semi-local FCHL achieves an MAE of 5.67 kcal/mol, which is slightly better than the global FCHL and shows a clear improvement over the local FCHL of the metal center.

Table S14: Prediction accuracy for spin-splitting energies ($\Delta E_{\mathrm{HS-LS}}$, in kcal/mol) evaluated separately on the TM-GSspin^{+} subsets defined by the sign of $\Delta E_{\mathrm{HS-LS}}$: $\Delta E_{\mathrm{HS-LS}} < 0$ (1,335 complexes) and $\Delta E_{\mathrm{HS-LS}} > 0$ (925 complexes). MAE denotes the mean absolute error, and STD denotes the standard deviation across the folds in 10-fold cross-validation. For ε-SPA^{H}M, the spin state used to construct the representation is indicated in parentheses as ground-state spin (GS), low-spin (LS), or high-spin (HS). For SPA^{H}M(a) and SPA^{H}M(b), only the ground-state spin is used for both local and global variants.

	$\Delta E_{\mathrm{HS-LS}} < 0$		$\Delta E_{\mathrm{HS-LS}} > 0$	
method	MAE	STD	MAE	STD
		Global		
SLATM	7.23	0.59	10.27	0.76
FCHL	8.06	0.63	11.97	1.78
SOAP	7.75	0.60	11.54	1.41
ε-SPA^{H}M (GS)	10.27	0.69	19.31	1.38
ε-SPA^{H}M (LS)	10.34	0.75	19.36	1.44
ε-SPA^{H}M (HS)	10.30	0.68	19.34	1.40
SPA^{H}M(a)	8.33	0.53	11.94	1.80
SPA^{H}M(b)	8.08	0.87	9.88	1.09
		Local		
aSLATM	6.46	0.46	9.61	0.97
FCHL	10.27	0.55	10.64	1.38
SOAP	6.85	0.44	9.72	1.40
SPA^{H}M(a)	6.83	0.60	8.53	0.72
SPA^{H}M(b)	8.21	0.70	10.28	1.14

Table S15: Mean absolute errors on the Octa-MK validation set, which follows the 80/20 training/validation split used in the original study by Meyer *et al.*[10] The target properties include the spin-splitting energy (in kcal/mol) and the HOMO, LUMO, and HOMO–LUMO gap (in eV). For MAEs in spin-splitting energy, both global (entire complex) and local variants (metal center) are reported where applicable. The best-performing models evaluated in this work are highlighted in bold. For comparison, MAEs of the KRR and NN models (standard-RACs, two-body, and three-body) reported in Meyer *et al.*[10] are also included, and the best-performing model in their work is also highlighted in bold.

method	Spin-splitting energy		HOMO	LUMO	HOMO–LUMO gap
	Global	Local			
SLATM	5.19	4.96	1.31	1.45	0.51
FCHL	6.84	11.50	2.01	2.32	0.74
SOAP	6.01	6.92	1.82	2.10	0.69
ε-SPA^{H}M	9.25	–	0.51	0.69	0.64
SPA^{H}M(a)	4.63	4.06	1.79	1.70	0.50
SPA^{H}M(b)	5.69	6.10	1.43	1.67	0.66
3DMol	4.58	4.88	1.90	1.69	0.60
MACE	6.11	–	1.44	1.62	0.56
3DMol-QS	**3.77**	**3.47**	0.51	0.85	0.46
MACE-QS	3.82	–	**0.32**	**0.30**	**0.33**
KRR standard-RACs	3.67		0.37		0.52
KRR two-body	3.96		0.44		0.45
KRR three-body	**3.30**		0.40		0.41
NN standard-RACs	3.51		**0.34**		0.43
NN two-body	3.73		0.43		0.44
NN three-body	3.48		0.38		**0.39**

Table S16: Comparison of the mean absolute error in predicting spin-splitting energies (in kcal/mol) in Octa-MK for molecular representations constructed using HS- or LS-optimized geometries. For quantum-informed SPA^{H}M representations, four input configurations are evaluated: LS-optimized geometry with LS, LS-optimized geometry with the lowest-energy spin state, HS-optimized geometry with HS, and HS-optimized geometries with the lowest-energy spin state.

		Input used to construct representations	
Representation	Spin	HS-optimized geometry	LS-optimized geometry
Global			
SLATM	–	5.03	4.62
FCHL	–	6.40	6.09
SOAP	–	5.48	5.14
ε-SPA^{H}M	LS	8.20	7.69
	lower-energy state spin	7.64	7.77
SPA^{H}M(a)	LS	4.83	3.98
	lower-energy state spin	4.56	4.45
SPA^{H}M(b)	LS	5.04	4.37
	lower-energy state spin	4.13	4.11
Local			
aSLATM	–	5.07	4.39
FCHL	–	12.16	10.47
SOAP	–	5.48	5.19
SPA^{H}M(a)	LS	4.65	3.60
	lower-energy state spin	4.09	3.83
SPA^{H}M(b)	LS	6.47	5.55
	lower-energy state spin	4.78	4.45

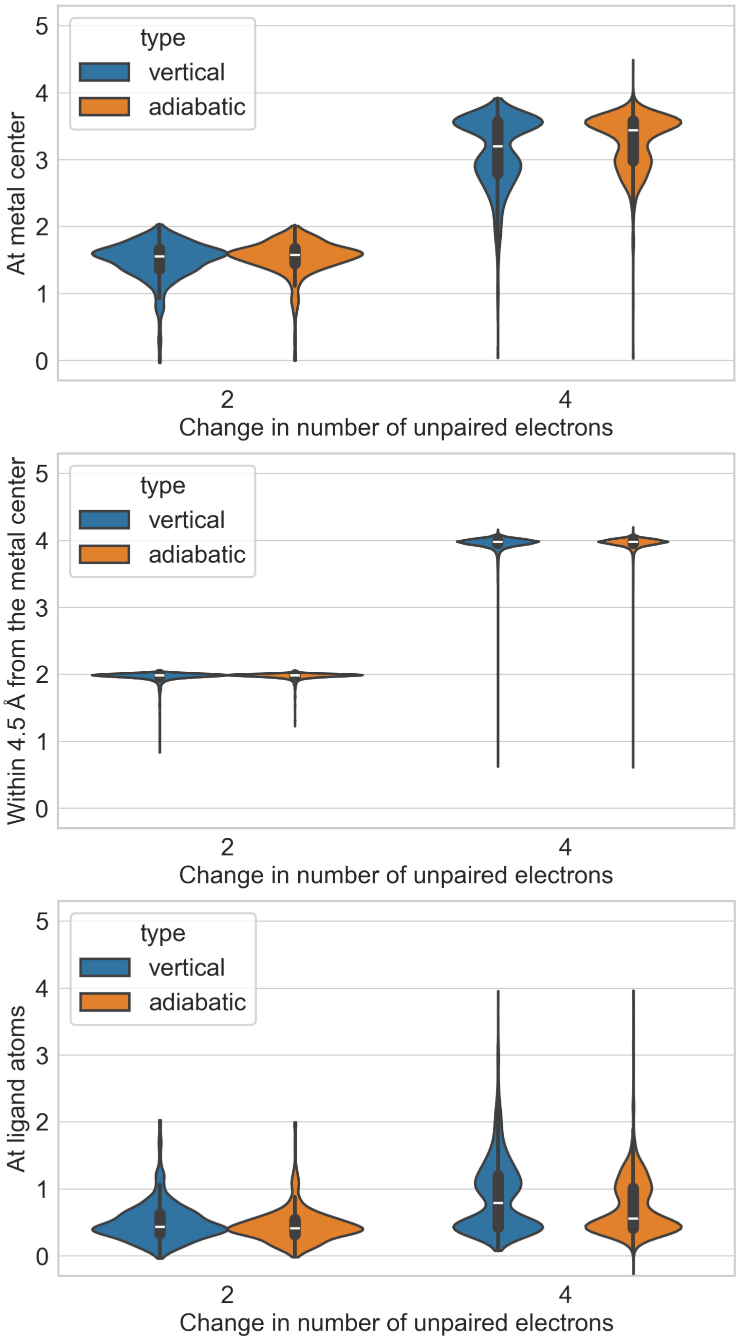

Figure S1: Violin plots of Hirshfeld spin-population changes associated with the LS-to-HS transition in TM-GSspin$^+$ complexes, grouped by the change in the number of unpaired electrons, for vertical and adiabatic computations. The panels report spin-population changes at the metal center (top), the sum over atoms within 4.5 Å of the metal center (middle), and the sum over all ligand atoms (bottom).

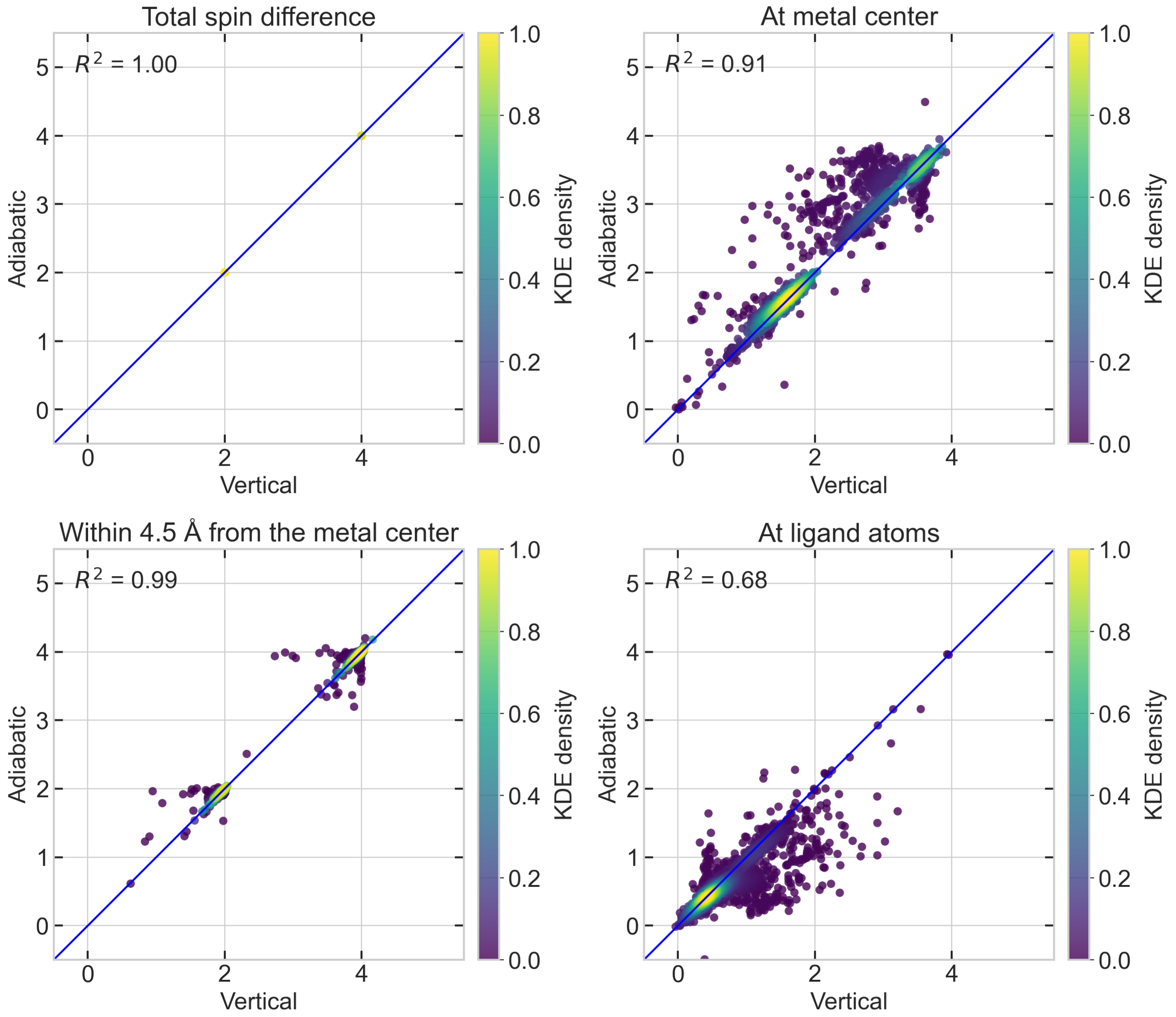

Figure S2: Hirshfeld spin-population differences associated with the LS-to-HS transition in TM-GSspin$^+$ complexes, comparing vertical and adiabatic computations. The panels show the total spin difference (top left), the contribution at the metal center (top right), the cumulative contribution from atoms within 4.5 Å of the metal center (bottom left), and the contribution at ligand atoms (bottom right). Point colors indicate kernel density estimate (KDE) density, the blue line marks perfect agreement, and R^2 values quantify the correlation between the two types of computations.

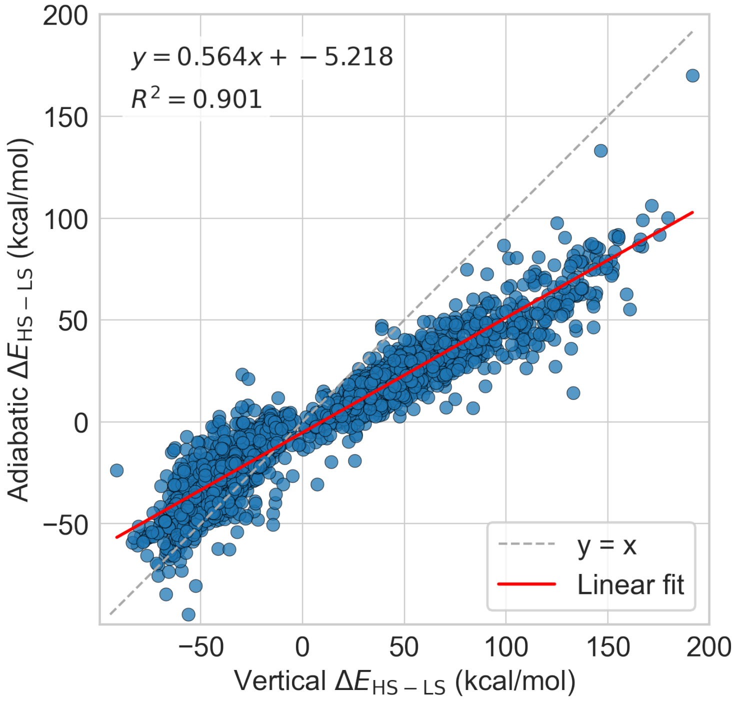

Figure S3: Correlation plot between vertical and adiabatic spin-splitting energies of TM-GSspin$^+$, computed at the B3LYP*-D3(BJ)/def2-TZVP level. Fifteen complexes were excluded because full geometry optimization did not converge for all accessible spin states. One complex, $[FeCl_6]^{4-}$, was excluded since two chloride ligands dissociated and moved away from the metal center during geometry optimization in the HS state.

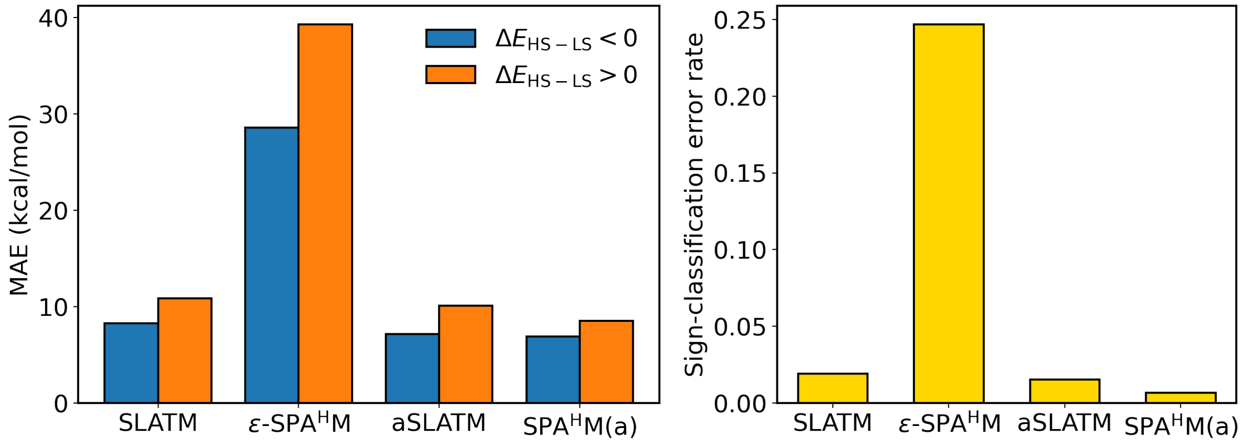

Figure S4: Performance breakdown of KRR models on the TM-GSspin$^+$ for predicting spin-splitting energies. Mean absolute errors (MAEs, in kcal/mol) are evaluated using 10-fold cross-validation on the full dataset and grouped by the sign of the reference $\Delta E_{\text{HS-LS}}$. **Left:** MAEs for subsets with $\Delta E_{\text{HS}-\text{LS}} < 0$ (blue) and $\Delta E_{\text{HS}-\text{LS}} > 0$ (orange) of four KRR models using SLATM, ε-SPA^{H}M, aSLATM, and SPA^{H}M(a). **Right:** Sign-classification error rate for the HS/LS energetic ordering, defined as the fraction of predictions with the wrong sign of $\Delta E_{\text{HS}-\text{LS}}$.

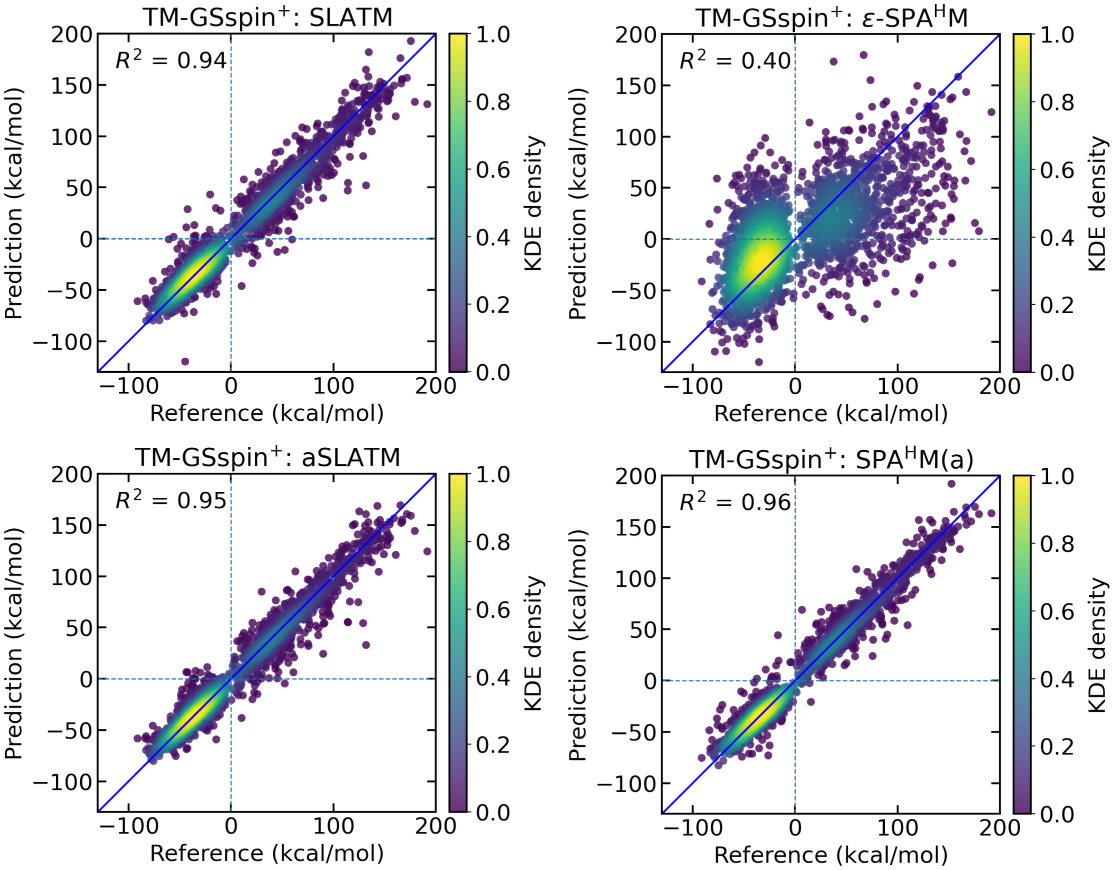

Figure S5: Parity plots comparing ML-predicted and DFT reference spin-splitting energies (kcal/mol) for the TM-GSspin$^+$ using KRR models with four representations: SLATM (top left), ε-SPA^{H}M (top right), aSLATM (bottom left), and SPA^{H}M(a) (bottom right). Point colors indicate the KDE-estimated data density.

S3.2 Frontier molecular orbital energies

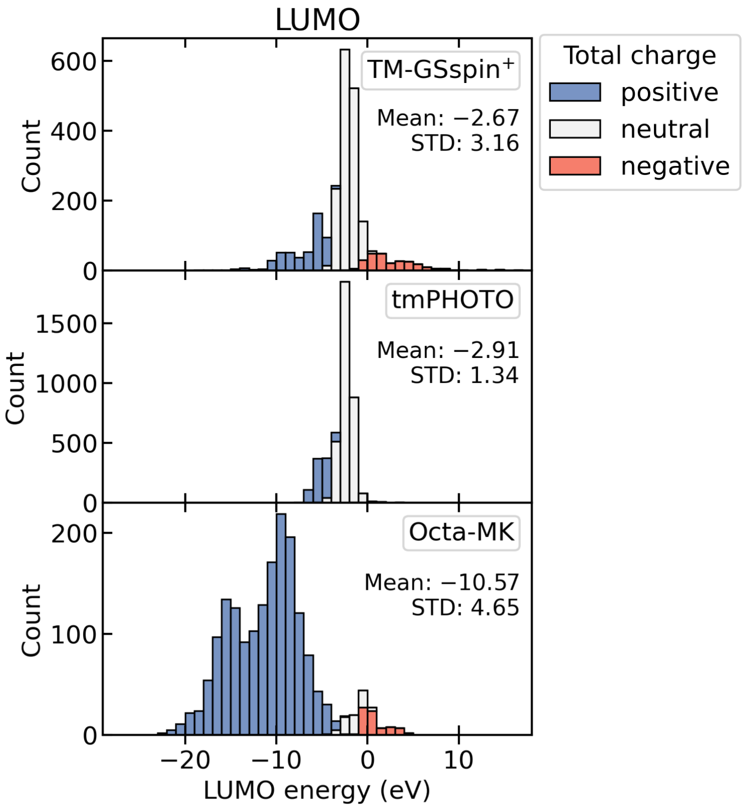

Figure S6: Stacked histograms of LUMO distributions for the benchmark datasets: TM-GSspin$^+$ (top), tmPHOTO (middle), and Octa-MK (bottom). Colors represent the total charge (blue: positive, white: neutral, red: negative).

Table S17: Prediction accuracy for HOMO, LUMO, and HOMO–LUMO gap across the datasets. MAE denotes the mean absolute error, and STD refers to the standard deviation across the folds in 10-fold cross-validation.

(a) HOMO (eV)

	TM-GSspin$^+$		tmPHOTO		Octa-MK	
method	MAE	STD	MAE	STD	MAE	STD
SLATM	1.02	0.08	0.33	0.02	0.74	0.06
FCHL	1.36	0.07	0.52	0.03	1.06	0.08
SOAP	1.27	0.08	0.44	0.03	1.03	0.09
ε-SPA^{H}M	0.61	0.04	0.32	0.01	0.40	0.03
SPA^{H}M(a)	1.50	0.22	0.56	0.03	1.04	0.09
SPA^{H}M(b)	0.73	0.09	0.32	0.03	0.51	0.05
3DMol	1.23	0.09	0.31	0.02	0.87	0.13
MACE	1.24	0.11	0.30	0.04	1.07	0.11
3DMol-QS	0.43	0.03	0.18	0.01	0.26	0.02
MACE-QS	0.35	0.04	0.16	0.01	0.21	0.01

(b) LUMO (eV)

	TM-GSspin$^+$		tmPHOTO		Octa-MK	
method	MAE	STD	MAE	STD	MAE	STD
SLATM	1.07	0.08	0.34	0.01	0.85	0.06
FCHL	1.39	0.09	0.55	0.02	1.22	0.11
SOAP	1.27	0.09	0.46	0.02	1.17	0.11
ε-SPA^{H}M	0.74	0.05	0.42	0.01	0.52	0.04
SPA^{H}M(a)	1.59	0.15	0.60	0.02	1.00	0.12
SPA^{H}M(b)	0.78	0.10	0.36	0.02	0.55	0.08
3DMol	1.30	0.10	0.28	0.03	1.02	0.11
MACE	1.24	0.09	0.34	0.06	1.23	0.07
3DMol-QS	0.50	0.06	0.18	0.02	0.33	0.03
MACE-QS	0.40	0.06	0.19	0.01	0.20	0.03

(c) HOMO-LUMO gap (eV)

	TM-GSspin$^+$		tmPHOTO		Octa-MK	
method	MAE	STD	MAE	STD	MAE	STD
SLATM	0.38	0.03	0.21	0.01	0.45	0.03
FCHL	0.43	0.03	0.28	0.01	0.62	0.05
SOAP	0.40	0.03	0.24	0.01	0.55	0.03
ε-SPA^{H}M	0.54	0.02	0.47	0.01	0.57	0.02
SPA^{H}M(a)	0.45	0.03	0.32	0.01	0.45	0.03
SPA^{H}M(b)	0.42	0.03	0.30	0.02	0.43	0.03
3DMol	0.43	0.02	0.22	0.01	0.47	0.02
MACE	0.43	0.04	0.22	0.01	0.48	0.02
3DMol-QS	0.44	0.02	0.22	0.01	0.34	0.02
MACE-QS	0.36	0.03	0.22	0.01	0.25	0.02

Table S18: Mean absolute errors of graph neural network models based on NatQG graphs[11] for the test set of 2,696 tmPHOTO complexes. The u-NatQG and d-NatQG graph representations were evaluated using the adapted MPNN and MPNN⊕G architectures, following Kneiding *et al.*[11] These complexes are shared between tmPHOTO and tmQMg and account for $\sim 65\%$ of tmPHOTO. The models were trained from scratch using the authors' GitHub repository,[12] following the original training protocol with a random 80/10/10 train/validation/test split and the optimized hyperparameters reported therein. For comparison, the MAEs reported for the tmQMg test set in the original study are also included.

(a) HOMO-LUMO gap (mHa)

Architecture	Graph	65% of tmPHOTO	tmQMg
MPNN	u-NatQG	6.72	6.02
	d-NatQG	7.48	7.22
MPNN⊕G	u-NatQG	6.90	6.04
	d-NatQG	6.96	7.19

(b) Dipole moment (Debye)

Architecture	Graph	65% of tmPHOTO	tmQMg
MPNN	u-NatQG	1.782	0.819
	d-NatQG	1.645	1.019
MPNN⊕G	u-NatQG	1.915	0.895
	d-NatQG	1.762	0.981

Figure S7 presents the HOMO distributions for each charge subset in TM-GSspin$^+$ and tmPHOTO.

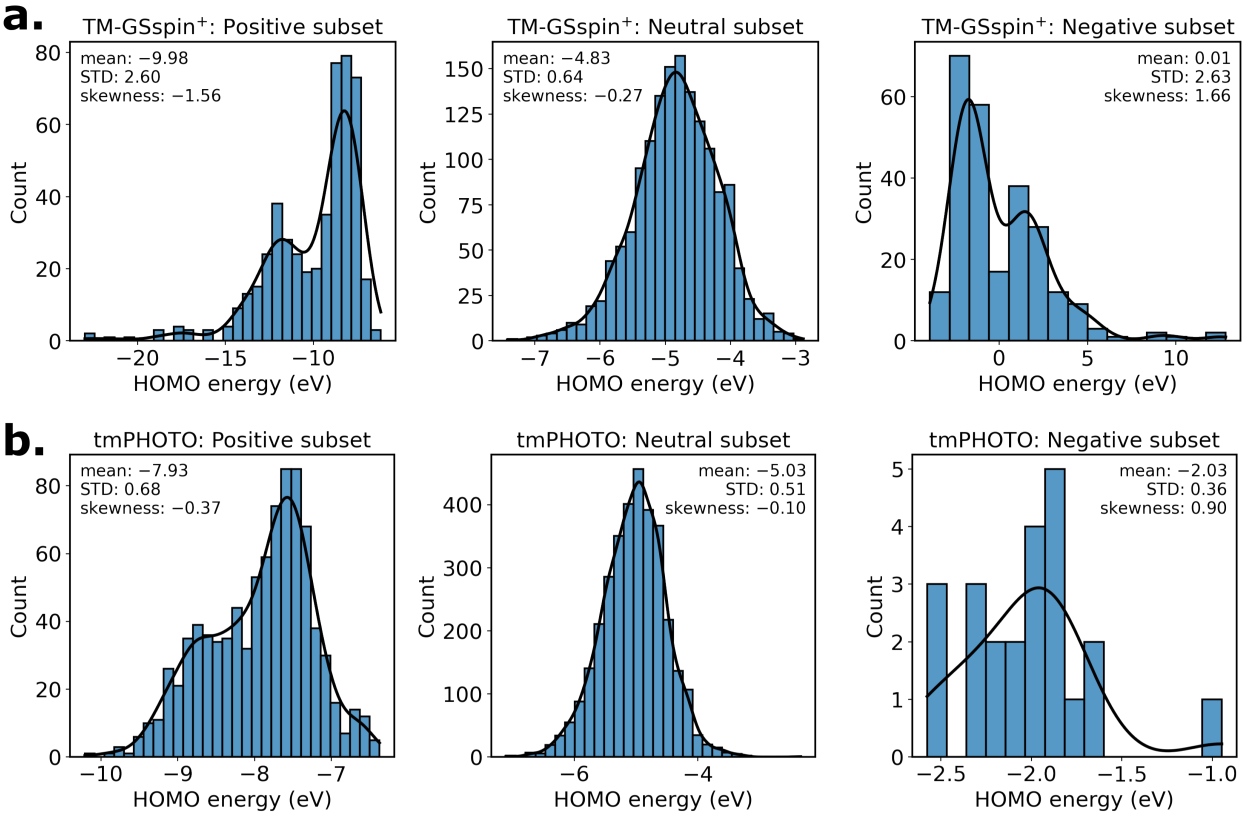

Figure S7: HOMO energy distributions for positive (left), neutral (center), and negative (right) subsets in **a.** TM-GSspin$^+$ and **b.** tmPHOTO. The inset shows the mean, standard deviation (STD), and skewness (with larger absolute values indicating stronger skewness).

The HOMO distributions of the neutral subsets are symmetric in both TM-GSspin$^+$ and tmPHOTO (Figure S7**a** and **b**, center). In TM-GSspin$^+$, however, the positive and negative subsets exhibit strong skewness (Figure S7**a**, left and right). This skewness leads to large within-subset MAEs for both SLATM and ε-SPA^{H}M, since kernel models tend to overfit dense regions and fail to learn sparse tails. In contrast, the overall HOMO distribution of TM-GSspin$^+$ is less skewed, enabling better generalization across charge when models are trained on the full dataset.

In tmPHOTO, the positively charged subset is only mildly skewed (Figure S7**b**, left), reducing the learning difficulty during subset-restricted training. Although the HOMO ranges

of the positive and neutral subsets differ, both distributions are nearly symmetric. As a result, SLATM achieves lower within-subset MAEs than those from the full dataset, which is dominated by neutral species. In contrast, ε-SPA^{H}M does not show this improvement, suggesting that its performance is already saturated given the dataset size and property distribution.

Figure S8 presents the HOMO–LUMO gap distributions for the individual charge subsets in TM-GSspin$^+$ and tmPHOTO. In TM-GSspin$^+$, the gap distributions of the positive and negative subsets (Figure S8**a**, left and right) are considerably more symmetric than the corresponding HOMO distributions, which allows the KRR models to learn the gap more effectively within each subset.

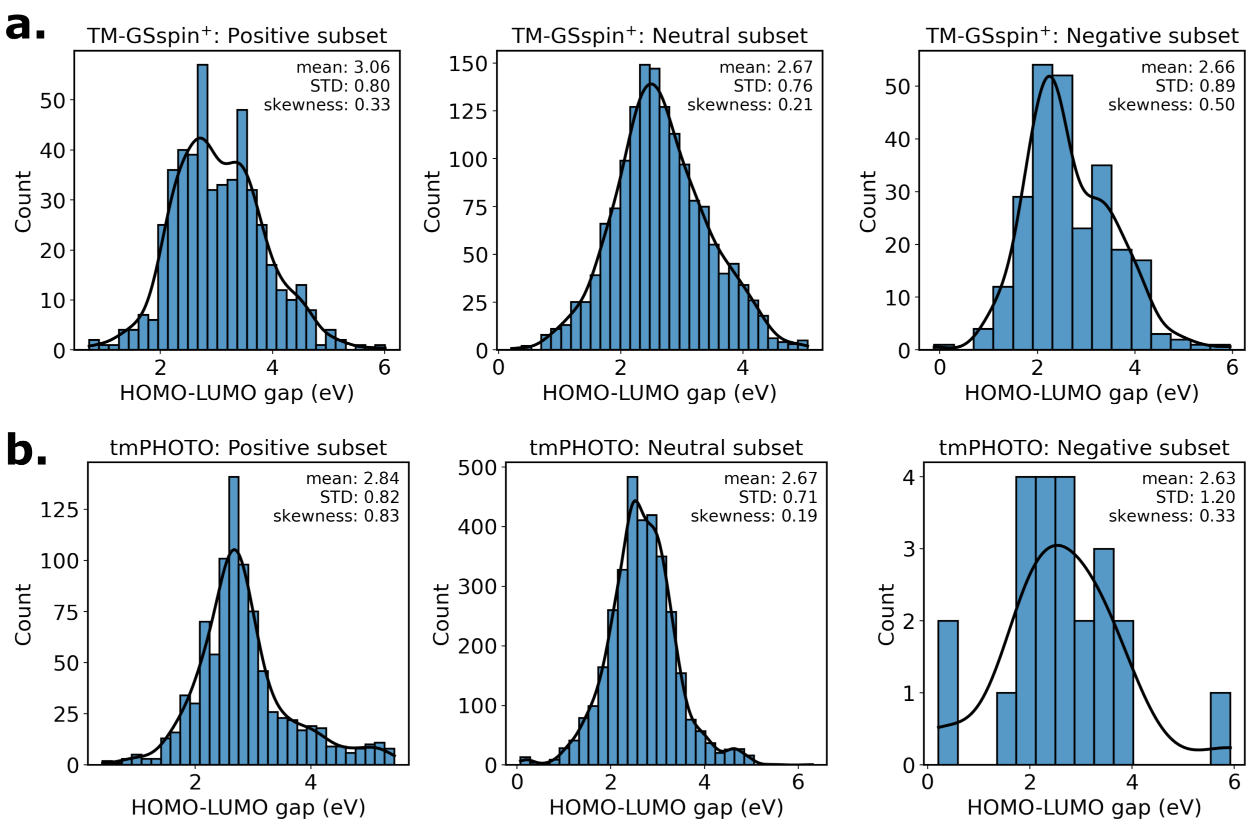

Figure S8: HOMO-LUMO gap distributions for positive (left), neutral (center), and negative (right) subsets in **a.** TM-GSspin$^+$ and **b.** tmPHOTO. The inset shows the mean, standard deviation (STD), and skewness (with larger absolute values indicating stronger skewness).

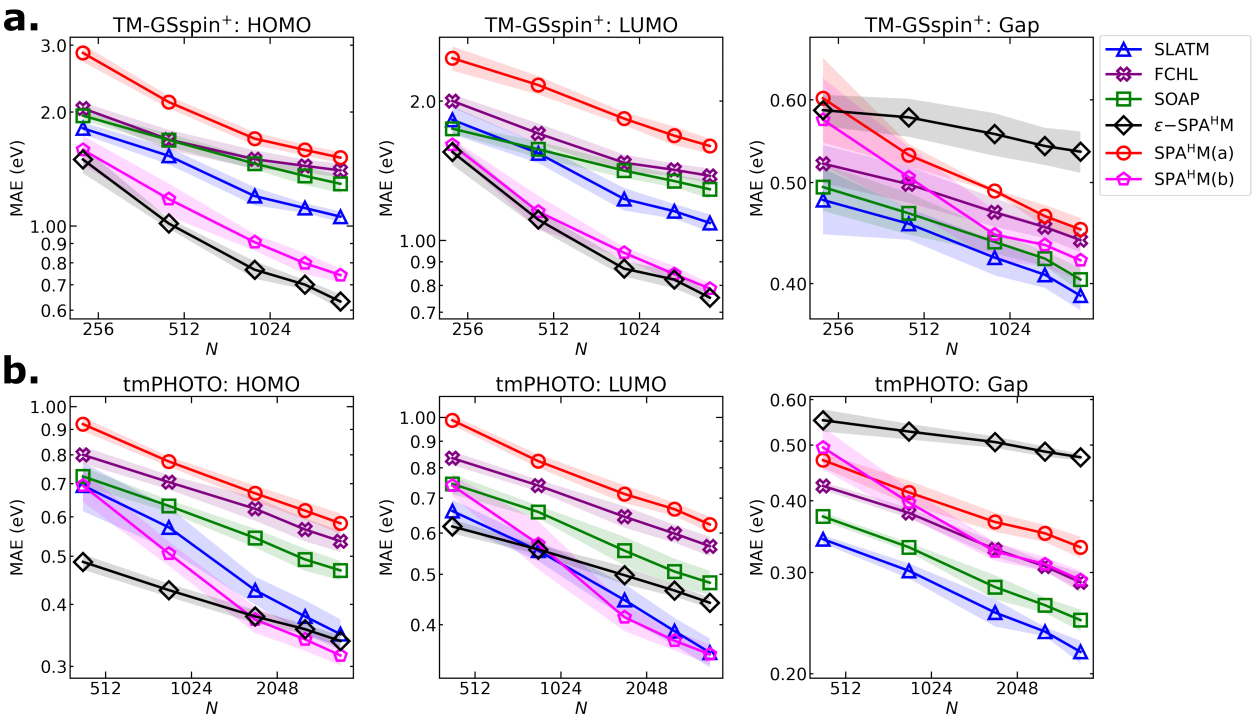

Figure S9: Learning curves of KRR models using different molecular representations to predict HOMO (left), LUMO (center), and HOMO–LUMO gap (right) for **a.** TM-GSspin$^+$ and **b.** tmPHOTO.

S3.3 Dipole moment

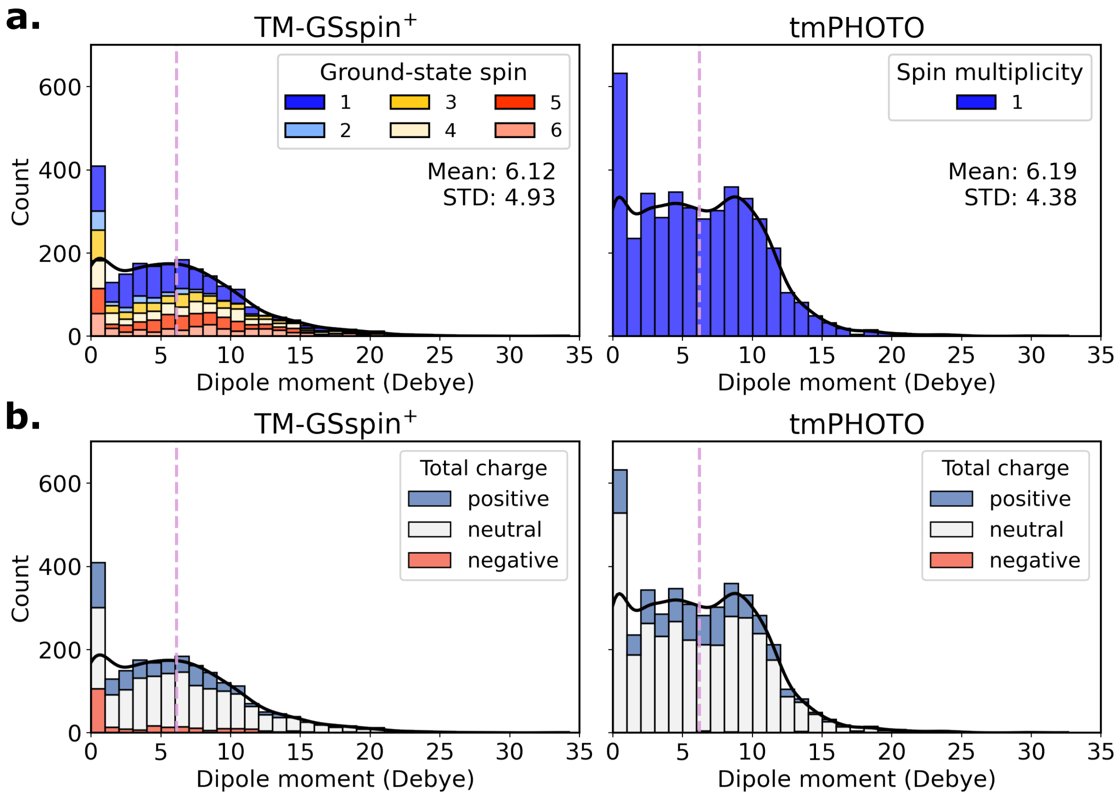

Figure S10: Distribution of dipole moment magnitudes for TM-GSspin$^+$ (left) and tmPHOTO (right), shown as stacked histograms. The dashed line indicates the mean value. **a.** Colors represent the spin multiplicities used to compute the property. **b.** Colors represent the total charge of the complexes (blue: positive, white: neutral, red: negative).

Table S19: Prediction accuracy for dipole moment magnitudes across the datasets. MAE denotes the mean absolute error, and STD refers to the standard deviation across the folds in 10-fold cross-validation.

	TM-GSspin$^+$		tmPHOTO	
method	MAE	STD	MAE	STD
SLATM	2.42	0.12	1.53	0.09
FCHL	2.44	0.08	1.81	0.10
SOAP	2.20	0.10	1.60	0.10
ε-SPA^{H}M	3.45	0.15	2.83	0.11
SPA^{H}M(a)	3.06	0.13	2.19	0.10
SPA^{H}M(b)	2.83	0.10	2.00	0.13
3DMol	1.97	0.21	1.60	0.09
MACE (`AtomicDipolesMACE`, equi.)	1.60	0.13	1.05	0.06
3DMol-QS	2.13	0.18	1.62	0.09

For exploratory comparison, we also evaluate the MACE models originally developed for energy prediction, in both their invariant and equivariant forms. These architectures are designed to predict global scalar energies within the `MACE` architecture and are not intended for dipole moment prediction. In principle, applying them would require converting the units to eV, as recommended by the developers; however, for this exploratory single-fold evaluation on TM-GSspin$^+$ (rather than full 10-fold CV), we retained the Debye units. These energy-targeting models perform substantially worse than the `AtomicDipolesMACE` model, and adding electronic information (charge, spin, or both) further degrades performance. This confirms that such architectures are unsuitable for predicting dipole-moment magnitudes, even though the final target is a scalar.

Table S20: Mean absolute errors (in Debye) for dipole-moment magnitude prediction on TM-GSspin$^+$ using an 80/10/10 train/validation/test split. Energy-targeting MACE models are evaluated in their invariant (in.) and equivariant (equi.) forms, with additional charge (–Q), spin (–S), or charge–spin (–QS) embeddings.

Exploratory comparison for MACE	TM-GSspin$^+$
MACE (`MACE`, in.)	1.78
MACE (`MACE`, equi.)	1.80
MACE-Q (`MACE`, in.)	2.57
MACE-Q (`MACE`, equi.)	2.31
MACE-S (`MACE`, in.)	2.74
MACE-S (`MACE`, equi.)	2.47
MACE-QS (`MACE`, in.)	2.58
MACE-QS (`MACE`, equi.)	2.39

S3.4 Timings

Table S21: Elapsed times in seconds for generating representations ("repr.") and computing the Laplacian kernel ("kernel") for subsets of TM-GSspin$^+$ and tmPHOTO, each containing 500 randomly selected complexes. The column labeled "sum" reports the combined time for representation generation and kernel computation, and "repr. size" column denotes the size of the respective representations (the number of features). All values are averaged over five subsets for each dataset.

	subset of TM-GSspin$^+$				subset of tmPHOTO			
method	repr.	kernel	sum	repr. size	repr.	kernel	sum	repr. size
Global								
SLATM	1489	93.9	1582.9	398,321	6141	248.4	6389.4	1,009,514
FCHL	982	1.6	983.6	7,272	1143	12.4	1155.4	13,600
SOAP	45	23.0	68.0	103,680	101	45.0	146.0	200,000
ε-SPA^{H}M	1187	0.1	1187.1	736	2805	0.2	2805.2	902
Local								
aSLATM	869	93.7	962.7	398,321	4258	249.3	4507.3	1,009,514
SOAP	8	23.0	31.0	103,680	14	44.8	58.8	200,000
SPA^{H}M(a)	34200	3.4	34203.4	15,342	77288	5.4	77293.4	19,980
SPA^{H}M(b)	20091	2.2	20093.2	9,972	43695	4.6	43699.6	13,850

Table S22: Elapsed times (in seconds) for generating the representations and computing the Gaussian kernel for subsets of the TM-GSspin$^+$ and tmPHOTO, each subset containing randomly chosen 500 complexes. The "sum" column represents the total time for generation of representations (column "repr.") and Gaussian kernels ("kernel"), while the "repr. size" column denotes the size of the respective representations (the number of features). All values are averaged over five subsets for each benchmark dataset.

	subset of TM-GSspin$^+$				subset of tmPHOTO			
method	repr.	kernel	sum	repr. size	repr.	kernel	sum	repr. size
Global								
SLATM	1489	3.5	1492.5	398,321	6141	8.8	6149.8	1,009,514
FCHL	982	0.1	982.1	7,272	1143	9.6	1152.6	13,600
SOAP	45	0.9	45.9	103,680	101	1.7	102.7	200,000
ε-SPA^{H}M	1187	0.0	1187.0	736	2805	0.0	2805.0	902
Local								
aSLATM	869	3.4	872.4	398,321	4258	8.7	4266.7	1,009,514
SOAP	8	0.9	8.9	103,680	14	1.7	15.7	200,000
SPA^{H}M(a)	34200	0.2	34200.2	15,342	77288	1.3	77289.3	19,980
SPA^{H}M(b)	20091	0.1	20091.1	9,972	43695	1.5	43696.5	13,850

Table S23: Elapsed times in seconds for 3DMol on TM-GSspin$^+$ and tmPHOTO. The column labeled "sum" reports the total time for initialization ("init"), training for 128 epochs ("train"), and test-set evaluation ("test") for HOMO–LUMO gap prediction. The "repr. size" column lists the dimensionality of the learned representation. Reported timings are averaged over five random splits from the 10-fold CV for each dataset.

	Full TM-GSspin$^+$ (2,260)					Full tmPHOTO (4,268)				
method	init	train	test	sum	repr. size	init	train	test	sum	repr. size
3DMol	31	167	0.5	198	64	98	320	0.7	419	64

TOC graphic

The molecular structures shown in the TOC graphic were rendered using xyzrender.[13]

References

(1) Vela, S.; Laplaza, R.; Cho, Y.; Corminboeuf, C. cell2mol: encoding chemistry to interpret crystallographic data. *npj Comput. Mater.* **2022**, *8*, 188.

(2) Cho, Y.; Laplaza, R.; Vela, S.; Corminboeuf, C. Automated prediction of ground state spin for transition metal complexes. *Digit. Discov.* **2024**, *3*, 1638–1647.

(3) Khan, D.; Heinen, S.; von Lilienfeld, O. A. Kernel based quantum machine learning at record rate: Many-body distribution functionals as compact representations. *J. Chem. Phys.* **2023**, *159*, 034106.

(4) Khan, D.; von Lilienfeld, O. A. Generalized convolutional many-body distribution functional representations. *Proc. Natl. Acad. Sci. U.S.A.* **2025**, *122*, e2415662122.

(5) Santiago, R.; Vela, S.; Deumal, M.; Ribas-Arino, J. Unlocking the predictive power of quantum-inspired representations for intermolecular properties in machine learning. *Digit. Discov.* **2024**, *3*, 99–112.

(6) Van Lenthe, J. H.; Zwaans, R.; Van Dam, H. J. J.; Guest, M. F. Starting SCF calculations by superposition of atomic densities. *J. Comput. Chem.* **2006**, *27*, 926–932.

(7) Llenga, S.; Gryn'ova, G. Matrix of orthogonalized atomic orbital coefficients representation for radicals and ions. *J. Chem. Phys.* **2023**, *158*, 214116.

(8) Lopanitsyna, N.; Fraux, G.; Springer, M. A.; De, S.; Ceriotti, M. Modeling high-entropy transition metal alloys with alchemical compression. *Phys. Rev. Mater.* **2023**, *7*, 045802.

(9) van Gerwen, P.; Briling, K. R.; Bunne, C.; Somnath, V. R.; Laplaza, R.; Krause, A.; Corminboeuf, C. 3DReact: Geometric deep learning for chemical reactions. *J. Chem. Inf. Model.* **2024**, *64*, 5771–5785.

(10) Meyer, R.; Chu, D. B.; Kulik, H. J. Many-body expansion based machine learning models for octahedral transition metal complexes. *Mach. Learn.: Sci. Technol.* **2025**, *5*, 045080.

(11) Kneiding, H.; Lukin, R.; Lang, L.; Reine, S.; Pedersen, T. B.; De Bin, R.; Balcells, D. Deep learning metal complex properties with natural quantum graphs. *Digit. Discov.* **2023**, *2*, 618–633.

(12) Kneiding, H.; Lukin, R. tmQMg: Repository for the tmQMg dataset files and analysis scripts. `https://github.com/uiocompcat/tmQMg`, 2024.

(13) Goodfellow, A.; Nguyen, B. Graph-Based Internal Coordinate Analysis for Transition State Characterization. *J. Chem. Theory Comput.* **2026**, *22*, 2348–2357.
